# Burden of severe maternal morbidity and association with adverse birth outcomes in sub–Saharan Africa and south Asia: protocol for a prospective cohort study

**DOI:** 10.7189/jogh.6.020601

**Published:** 2016-12

**Authors:** AMANHI Maternal Morbidity study group

**Affiliations:** AMANHI – Alliance for Maternal and Newborn Health Improvement

## Abstract

**Objectives:**

The AMANHI morbidity study aims to quantify and describe severe maternal morbidities and assess their associations with adverse maternal, fetal and newborn outcomes in predominantly rural areas of nine sites in eight South Asian and sub–Saharan African countries.

**Methods:**

AMANHI takes advantage of on–going population–based cohort studies covering approximately 2 million women of reproductive age with 1– to 3–monthly pregnancy surveillance to enrol pregnant women. Morbidity information is collected at five follow–up home visits – three during the antenatal period at 24–28 weeks, 32–36 weeks and 37+ weeks of pregnancy and two during the postpartum period at 1–6 days and after 42–60 days after birth. Structured–questionnaires are used to collect self–reported maternal morbidities including hemorrhage, hypertensive disorders, infections, difficulty in labor and obstetric fistula, as well as care–seeking for these morbidities and outcomes for mothers and babies. Additionally, structured questionnaires are used to interview birth attendants who attended women’s deliveries. All protocols were harmonised across the sites including training, implementation and operationalising definitions for maternal morbidities.

**Importance of the AMANHI morbidity study:**

Availability of reliable data to synthesize evidence for policy direction, interventions and programmes, remains a crucial step for prioritization and ensuring equitable delivery of maternal health interventions especially in high burden areas. AMANHI is one of the first large harmonized population–based cohort studies being conducted in several rural centres in South Asia and sub–Saharan Africa, and is expected to make substantial contributions to global knowledge on maternal morbidity burden and its implications.

Pregnancy, childbirth and their related complications present a high level of risk to the survival, health and well–being of women and their babies in low and middle income country settings. Maternal mortality is the most commonly cited maternal health statistic: approximately 289 000 women die annually from pregnancy–related causes, 85% of these deaths occur in sub–Saharan Africa and South Asia alone [1]. The major direct causes of maternal deaths include hemorrhage, infection, unsafe abortion, eclampsia and obstructed labor [1–3]. With each maternal death, however, an additional 20 to 30 women are estimated to suffer acute morbidity and disabilities with substantial impact on their physical, psychological, social and economic functioning [4–7]. An estimated 15% of all pregnant women, approximately 20 million women globally, suffer a spectrum of maternal illnesses ranging in severity from mild disease to acute severe, life–threatening complications or near death events [3,8–10]. The challenge is that maternal ill–health and its effects are not well defined and seldom measured [11]; estimates are imprecise and likely underestimate the true burden [2,6,11,12], thereby undermining efforts to harness resources to address them.

Most studies on maternal morbidity are facility–based and are conducted in developed countries. There are marked disparities between developing and developed countries with respect to the prevalence of maternal morbidities. This could be a function of the higher frequency of childbirth, poorer general health of women, lack of care seeking and low quality of care available in developing countries. There is also a suggestion that women from some geographic regions might carry relatively high risk of maternal morbidity even if their environment is improved. For instance, a multi–country study among migrant populations in Australia, Canada and Denmark, found that, compared to non–migrants, migrants from sub–Saharan Africa appeared to have higher maternal morbidity risks and these findings were consistent with findings from studies conducted in Italy, Belgium the Netherlands and the UK [13–18]. In low– and middle–income countries (LMICs, especially those in sub–Saharan Africa and South Asia where the burden of morbidity is largest), there is a dearth of data on morbidity because maternal access to facilities is poor and vital registration systems are lacking or incomplete. Facility–based data are not enough to describe the true burden of morbidities but data from the community level, where many births and pregnancy–associated complications occur, are also particularly lacking and the quality of reporting is often poor. Prevalence estimates for morbidities are based on statistical models with substantial uncertainties around them. There is a clear need to generate high quality and reliable population–based estimates of severe maternal morbidity using robust epidemiological methods especially in sub–Saharan Africa and Asia.

The Alliance for Maternal and Newborn Health Improvement (AMANHI) maternal morbidity study directly responds to this need. The study aims to describe and quantify severe maternal morbidities and assess their associations with adverse maternal, fetal and newborn outcomes. It is being implemented at nine sites in eight countries of sub–Saharan Africa and South Asia. The study uses harmonized methods to collect prospective population–level maternal morbidity data. AMANHI morbidity study, coordinated by the Maternal, Newborn, Child and Adolescent Health department of the World Health Organization (WHO/MCA) will contribute to improving estimates of severe maternal morbidity; provide a better understanding of the contributory factors that require consideration when designing interventions; and inform the focus of future interventions in order to optimize impact. This manuscript describes the protocol for the harmonized implementation of the study.

## OBJECTIVES

The objectives are to determine the burden of severe acute maternal morbidity, describe the care received by pregnant and delivered women, and examine the association of severe maternal morbidity and care received with adverse maternal, fetal and neonatal outcomes.

## METHODS

### Study design and setting

The AMANHI morbidity study is a population–based, prospective cohort study. Trained AMANHI morbidity study fieldworkers conduct routine surveillance home visits to identify pregnant women, enrol them for follow–up through the pregnancy till after 42 days postpartum to collect data on morbidity, care seeking and outcomes for mothers and babies including preterm birth, intrauterine growth restriction (IUGR), stillbirths and neonatal mortality. It is built on an existing platform of neonatal health studies being implemented in Bangladesh (Sylhet), India (Uttar Pradesh), Pakistan (Karachi and Matiari) in south Asia; and Democratic Republic of Congo (Equator), Ghana (Kintampo), Kenya (Western province), Tanzania (Pemba) and Zambia (Southern Province Zambia) in sub–Saharan Africa. The study spans a period of 24–36 months, with staggered implementation across sites, starting in 2013 and expected to end in 2016.

### Study population and setting

The AMANHI morbidity study is being implemented in predominantly rural populations where women’s educational levels are low. A summary of the characteristics of the study sites is as shown in **Table 1**. Families mainly engage in subsistence agriculture, petty trading and fishing. A variety of health facilities ranging from community clinics (providing only first aid and referral services) to district hospitals serve the population. In AMANHI, these health facilities were mapped according to their type (health post/community clinic, health center, district or provincial hospital) and range of services provided (out–patients only; basic delivery services; basic or comprehensive emergency obstetric care). This mapping was done as part of on–going community–based pregnancy and birth surveillance that involves 1– to 3–monthly household visits by trained fieldworkers to all women of reproductive age (15 to 49 years). The exception to the community surveillance is Zambia where recruitment is facility–based as explained below. With each woman visited at least once every three months, pregnancies are identified early and any complications or adverse outcomes are documented close to when they occur. Any woman of reproductive age who resides in the study area is eligible for enrolment into the study once they fall pregnant and consent to participate. In order to generate comparable data that will be amenable to pooled analyses, the implementation of the study is harmonised across sites as described in the following sections.

**Table 1:** Summary description of the parent studies, surveillance system, surveillance population and annual number of births at AMANHI sites

Site	Parent study title and objective	Existing pregnancy surveillance system	Total surveillance population	Reproductive–aged women in surveillance	Approximate annual births
Bangladesh	Etiology of Neonatal Infection in South Asia (ANISA): To estimate community level etiology–specific incidence predictive risk factors and clinical features, treatment and prevention strategies for serious infections among young infants (0–59 days).	2–monthly by trained community health workers (CHWs)	600 000	88 000	13 000
Democratic Republic of Congo (DRC)	African Neonatal Sepsis Trial (AFRINEST): to test the safety and efficacy of simplified antibiotic regimens for treating possible serious bacterial infection in 0–59 day–old infants	3–monthly by CHWs	699 288	65 000	12 000
Ghana	Neonatal vitamin A supplementation (NeovitA) study: to determine if vitamin A supplementation to neonates once, orally, <48 hours of birth will reduce neonatal, early and late infant mortality	Monthly by fieldworkers	700 000	147 000	21 000
India–Shivgarh	Topical emollient application to babies to prevent infection especially in preterms & ANISA studies	3–monthly by fieldworkers	1 350 000	184 430	44 000
Kenya	AFRINEST study: same as DRC	3–monthly by CHWs	400 000	30 000	10 000
Pakistan–Karachi	ANISA study: same as Bangladesh	3–monthly by fieldworkers	270 000	63 000	9500
Pakistan–Matiari	ANISA study: same as Bangladesh	3–monthly by fieldworkers	215 200	64 000	8000
Tanzania–Pemba	Chlorhexidine (CHX) study: to evaluate the efficacy of chlorhexidine cord cleansing on neonatal mortality	6 weekly by trained CHWs	390 000	72 000	14 000
Zambia	Chlorhexidine (CHX) study: to evaluate the efficacy of chlorhexidine cord cleansing on neonatal mortality	No pregnancy surveillance; facility ANC enrolment	25 000*	25 000	9000

*Zambia to recruit only from antenatal clinics.

### Harmonization of protocols and implementation strategies

When the study was planned in 2012, investigators from all participating sites agreed on common protocols, standard operating procedures, methods and strategies for implementation.

### Protocols

The principal investigators put together an agreed common protocol for the study. They developed an initial generic protocol from which all the sites developed specific adaptations for their sites. These protocols were submitted to and approved by ethical review committees of the WHO and at the respective sites.

### Standard operating procedures & implementing strategies

**Core variable tables.** The AMANHI investigators discussed common data to collect and collated these into a core variable table (CVT) to be used across sites (tables in the **Online Supplementary Document(Online Supplementary Document)**). The table specifies and defines signs and symptoms that are elicited during interviews with women, measurements, outcomes and important baseline variables such as maternal age, education, household assets and the format in which these data should be collected and stored across the sites (ie, as text, numeric or time/date formats). The variables on this table are included in questionnaires that were used across all sites.

**Figure 1:**
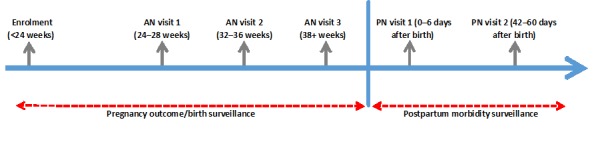
Antenatal (AN) and postnatal (PN) follow–up visit schedule–AMANHI morbidity study

**Timing and frequency of visits.** A uniform schedule for household visits by fieldworkers was used across AMANHI sites, as shown in **Figure 1**. Timing and frequency of the visits have been chosen to enable detailed information on women’s morbidity experiences, within each trimester of pregnancy, to be collected close to their occurrence. The first visit to the pregnant woman and her household is immediately after enrolment where fieldworkers conduct basic assets inventory and collect socio–demographic data. The first antenatal visit to collect morbidity information during pre–pregnancy and in early pregnancy prior to the visit is made at 24–28 weeks of pregnancy. Two more household visits are made during the antenatal period at 32–36 weeks and after 37 weeks of gestation to collect data on morbidities during the interval between the index and the previous visits and any care seeking around the pregnancy. Sites estimate gestational ages of pregnancies using women’s reported date of last normal menstrual period (LMP) to plan the antenatal visits. Two additional visits are made after delivery; within the first week (days 0–6) and after 42 days of birth to collect data on pregnancy outcomes, morbidities and their outcomes as well as care sought for the mother and baby. Measurements of blood pressure and urine proteins is made at each of the visits.

**Training of trainers for implementation.** The WHO/MCA trained and standardized AMANHI investigators from all the sites on the strategy for uniform implementation of the study across sites. The training involved the approach to consenting, collecting self–reported morbidity, measurement of blood pressure and testing urine for proteins for all participants in the study during household visits. Issues around confidentiality and sensitivity around eliciting unpleasant experiences from families were discussed. The complexity of measuring blood pressure within the home setting, collecting non–contaminated urine samples and ensuring accuracy of the measurements was particularly emphasized. Participants were trained to repeat all blood pressure (BP) measurements after 30 minutes of the initial recording. They discussed protocols for referral of women with abnormal findings (such as elevated BP and/or proteinuria) for appropriate care within health facilities. They were trained to conduct interviews with birth attendants and to review facility records in order to validate women’s reported morbidities. During practice sessions, participants followed the step–by–step process to practiced BP measurement on each other. Key areas of emphasis during training of fieldworkers in the sites were discussed.

**Training of data collectors at sites and quality assurance around data collection.** The investigators who participated in the harmonized training in turn trained study fieldworkers at their respective sites for the data collection. The study fieldworkers should have secondary/high school education (at least 10 years of formal education). The team also agreed on a common process for monitoring implementation and data quality across sites by the WHO/MCA. As part of the quality assurance protocol, ranges were specified for all variables and this was used to check the data. Data checks were done both on the field and in the data management centres within the respective sites. Inconsistent data are identified and rectified on the field, at the data centres or both. In addition, sites send cumulative study data to the WHO/MCA on a quarterly basis. Similar range and consistency checks are conducted on these data and outliers identified. The WHO data manager sends feedback to the sites for response and, where indicated, the WHO data set is updated accordingly. Trends in outliers and inconsistent data are analyzed according to the fieldworker who collected them and feedback sent to the sites to inform re–training of staff.

### Study supplies/equipment

Fieldworkers are provided equipment and training to directly assess pregnant women for hypertensive disorders during home visits. Each fieldworker uses urinalysis kits (Uristix^®^ by Siemens, Gujarat, India) to assess proteinuria and a digital sphygmomanometer (Microlife^®^ WatchBP^®^ Home A BP3MX1–3, Widnau, Switzerland) [19,20] to measure women’s blood pressure. All these study materials were procured from a common source.

### Surveillance for pregnancy identification

During home visits, fieldworkers use a variety of methods to identify pregnant women. These include direct disclosure by women or eliciting information on missed menstrual periods from women’s LMPs. When unsure, women in Bangladesh, Pakistan (Karachi and Matiari), India (UP) and Tanzania (Pemba) had the option to request a urine pregnancy test to confirm pregnancies. Zambia is the exception where, because over 96% of women in the study area attend antenatal care (ANC) clinics during pregnancy [21], recruitment into AMANHI is done at these ANC clinics. Once a woman is found to be pregnant, an information sheet containing a comprehensive summary of the study objectives, risks and benefits is read to potential participants in their local or their preferred language to help them make an informed decision to participate in the study. Consented mothers receive a unique study identification number (study ID).

### Follow–up on enrolled women

The AMANHI morbidity study employs both active and passive surveillance for collecting maternal morbidity data. In each site, fieldworkers use structured questionnaires (generated from the core variable table) to actively collect data on women’s self–reported morbidity and directly assess for hypertensive disorders during pregnancy and postpartum visits. As detailed in the next sub–section, questions on self–reported morbidity explored programmatically relevant morbidity causes such as antepartum hemorrhage, infections, miscarriage and abortions, signs and symptoms of hypertensive disorders of pregnancy such as severe headaches, blurred vision among others. For all births, fieldworkers also interview birth attendants (in the attendant’s home or place of work) to obtain additional details on complications each AMANHI study woman (whose delivery they assisted/attended) encounter during labor and delivery. Birth attendant account on morbidity will be used as validation for the women’s self–reported morbidity. The unique study ID provided to each enrolled woman links data from the two types of forms.

**Baseline home visit.** At baseline/enrolment fieldworkers collect household characteristics and baseline socio–demographic data on participants. They conduct an assets inventory which is used to classify households into socio–economic quintiles. This will be used to evaluate inequities in the distribution of severe maternal morbidity in the AMANHI cohort. The fieldworkers also collect data on previous medical and obstetric history, history of cigarette smoking or alcohol ingestion and morbidity experiences since the onset of the pregnancy.

**Listings for antenatal visits.** Women’s gestational age information is collected from either self–reported LMP or from any record of ultrasound scan conducted by the time of the visit. This information is used to estimate the gestational age of women. Each week, the study team generates a listing of women who are due for any of the antenatal visits and grouped into clusters that allow for fieldworkers to visit them for morbidity data collection. Whenever a woman is found to be temporarily away at the time of a scheduled visit, she is moved to the top of the listings for the next week and her visit is given priority and completed first.

**Antenatal home visits.** During the first antenatal home visit (conducted between 24–28 weeks), fieldworkers first ascertain the status of the pregnancy (whether woman is still pregnant or the pregnancy has terminated) and collect data on morbidity experiences. If the woman has commenced routine antenatal care (ANC) clinic attendance within the routine health system, fieldworkers abstract data on morbidity, results of any laboratory investigations (eg, hemoglobin level and presence of malaria parasites), ultrasound scans and maternal anthropometric measures (height, weight, mid upper arm circumference) taken by health professionals. At the end of the visit, they measure women’s BP and check their urine for proteins.

At subsequent antenatal home visits at 32–36 weeks and after 37 completed weeks, the same questionnaire is used to collect morbidity data covering the interval between the previous and the index visit.

The visit covers collection of reported morbidity and screening for hypertensive disorders in pregnancy. **Table 2** shows a summary of data collected at various visits in the AMANHI maternal morbidity study. Details on the process of data collection on each of the visit components are as follows:

**Table 2:** Summary of data collected at various visits in the AMANHI maternal morbidity study

Main category	Thematic areas of data collection	Source of data	Visit and time of data collection
Maternal morbidity	1. Antepartum hemorrhage	1. Maternal self–report	Antenatal home visits (24–28 weeks, 32–36 weeks, 37–40 weeks), postnatal home visits (day 1–6 and day 42–60 after birth), birth attendant interviews 0–6 days after birth, health facility records
	2. Postpartum hemorrhage	2. Maternal self–report and birth attendant interview	
	3. Hypertensive disorders of pregnancy	3. Measurements of blood pressure and urine protein at all home visits, maternal self–report	
	4. Difficulty in labor	4. Maternal self–report and birth attendant interview	
	5. Infection	5. Maternal self–report	
	6. Obstetric fistula	6. Maternal self–report	
Background characteristics	Socio–economic, baseline characteristics of the woman and her household, including an asset inventory	Maternal self–report	Baseline home visit at enrolment
Medical history	Previous obstetric and gynecological history, birth defects, prematurity, stillbirths and IUGR among previous babies, previous medical and surgical history	Maternal self–reports and health facility records	Baseline home visit at enrolment
Risk factors and exposures	Cigarette smoking, alcohol ingestion, smoke from biomass cooking fuels	Maternal self–reports	Baseline home visit at enrolment
Anthropometry	Paternal and maternal weights and heights, maternal mid–upper arm circumference	Health facility records	All antenatal and postnatal home visits
Screening for hypertensive disorders of pregnancy	Measurement of blood pressure and testing urine for proteins	Direct measurement during home visits	All visits except delivery visits

- **Reported morbidity.** Study fieldworkers ask questions around morbidities during the pregnancy using the study antenatal questionnaire derived from the core variable tables. These questions are to elicit any occurrence of severe maternal morbidity notably haemorrhage (antepartum and postpartum), infections, prolonged/obstructed labour, fistula, signs of pre–eclampsia or eclampsia. Since there are no current global standards for asking valid and reliable questions at the population level on maternal morbidity, AMANHI investigators agreed, pre–tested and validated questions to elicit maternal morbidities using scientific and pragmatic considerations of what data can be elicited from women at the community level. For example, for obstetric haemorrhage, the study used an adaptation of criteria suggested by Ronsmans [22] based on evidence of possible organ failure or life–saving surgical intervention. The limited evidence suggests that prolonged labour and postpartum haemorrhage (PPH) are particularly poorly reported. The standard definition of severity requires a quantification of the amount of blood lost (at least 500 ml for spontaneous vaginal delivery and 1000 ml for caesarean) to define PPH. In the home settings especially for deliveries that take place at home, it is difficult to quantify the amount of blood lost. AMANHI therefore used pragmatic definitions for these outcomes and prescribed these within the core variable table. In enquiring about severe PPH, AMANHI fieldworkers are trained to ask about any bleeding from the vagina after the birth of the baby, whether the bleeding was so much that it not only wet her clothes and the floor but also that the woman had to have an “operation” to stop it, she collapsed or lost consciousness as a result of or during the bleed. Similarly, for each morbidity included in the study, fieldworkers elicit information on the timing of onset, severity and any interventions received and from who this care was received.
- **Screening for hypertensive disorders of pregnancy.** Trained fieldworkers directly measure women’s BP and test their urine for proteins as part of active assessment for hypertensive disorders of pregnancy using a step–by–step protocol agreed across sites. The fieldworker first explains the rationale and procedure for the BP measurement and the urine sample testing. They make sure the woman sits and is made comfortable on a chair and the fieldworker places the digital sphygmomanometer on a surface at the level of her heart. Her blood pressure is then measured. If the pressure is found to be high, a repeat measurement is taken after 30 minutes of wait during which period the woman rests and is re–assured of the safety of the procedure. The blood pressure measurement is again repeated. To collect the urine sample, women are taught how to wipe her urethra with clean tissue and provided with a urine collection tube to obtain a sample of their urine for testing. Colorimetric methods are used to assess the degree of proteinuria coded from none through to 4 plus. At all visits, women with high blood pressure (systolic blood pressure >140 mm Hg or diastolic pressure >90 mm Hg) are referred to participating health facilities for appropriate care.

If the pregnancy has been aborted/miscarried, they terminate the AMANHI pregnancy follow–up and complete postnatal forms for the woman. At the first postnatal visit, data are collected on women’s reported morbidity during labor, delivery and immediately after birth including care seeking and outcomes for mother and baby. Fieldworkers also abstract morbidity data and the birthweight of babies from available health facility records (hospital folders, postnatal clinic record cards, etc.) during the postnatal visits.

Also following the realization of the difficulty in obtaining reliable data on complications such as hemorrhage through women’s self–reports, AMANHI uses an alternative source of data to corroborate women’s reported morbidity experiences during home visits – birth attendant accounts of morbidities during childbirth. These data are collected for all deliveries conducted by “professional” birth attendants (including traditional birth attendants, midwives, nurses and doctors).

**Birth attendant interviews.** Within the first week after every birth, fieldworkers identify and interview all delivery attendants in health facilities or who assisted five or more AMANHI deliveries (whether trained health professional or untrained traditional birth attendant – TBA). These interviews are done using structured questionnaires to provide more detail on women’s morbidity experiences (including complications) during labor, delivery and the immediate postpartum while under the care of the attendant. These interviews are held at the attendants’ home or place of work.

**Health facility records review**. In a few of the sites, data are collected, using a structured questionnaire, on the subset of women who attend health facilities for care during pregnancy, childbirth or in the postpartum period and used to validate morbidities from women’s and birth attendants’ reports. During home visits, data on premature births, intra–uterine growth retardation (IUGR) and mortality outcomes are also collected from this cohort.

**Verbal Autopsies for deaths**. Protocols for the conducting VAs in AMANHI are being published concurrently [23]. In summary, fieldworkers conduct verbal autopsies (VAs) whenever a woman of reproductive age, her fetus or a neonate dies using standardized tools and procedures. Trained field supervisors administer a verbal autopsy tool that has been developed using the WHO verbal autopsy tool as template. Additional questions were added on from tools used in other computer–based VA software available at the time. The supervisors obtain a narrative on the circumstances leading to the death, administer a semi–structured questionnaire to probe for specific signs and symptoms according to physiological systems and abstract data from any existing records including death certificates to help ascertain the type of death (eg, pregnancy–related or not, neonatal death or stillbirth), timing and the cause. Harmonised protocols are used by physicians who are selected from the respective countries and trained to confirm timing, type and to assign causes of these deaths based on principles of the International Classification of Diseases.

### Outcomes

The main outcome of the study is the prevalence of severe acute maternal morbidity (operationally defined to include acute problems suffered during pregnancy, through childbirth to the end of 42 days postpartum). Severe acute maternal morbidity will include, but is not limited to, pre–(eclampsia), antepartum and postpartum hemorrhage, abortion complications, maternal infections, obstructed labor and other complications arising out of these. Denominators for rate estimates will be total pregnancies or the number of women who become pregnant among the cohort while those who suffer any severe acute morbidity will contribute data to the numerators. In estimating prevalence of hypertensive disorders for which AMANHI is directly assessing women’s blood pressure and urine proteins at baseline (pre–pregnancy levels) and after 42 days postpartum (when those who developed pregnancy–induced hypertension will have returned to baseline states), it will be possible to describe a wide spectrum of hypertensive disorders including the classical pregnancy induced hypertension where women are normotensive pre–pregnancy, develop pregnancy–induced hypertension and return to normotensive state after delivery. Care seeking and care given for each morbidity will be described.

### Sample size considerations

The sample size contributions from each of the sites are as shown in **Table 3**. The 160 000 total participants in the study are sufficient for assessing association of severe maternal morbidity with adverse maternal, fetal and neonatal outcomes based on an assumption that all individual sites should have adequate power to detect association between preterm birth and any morbidity with a prevalence of 7.5% or more. Data will be pooled across sites for evaluating morbidities with lower prevalence, especially in assessing associations with stillbirths and early neonatal deaths.

**Table 3:** Expected number of participants to be enrolled from the AMANHI sites (by region) and precision that can be obtained around estimates

Region	Study country	Sample size	Expected width of 95% CI if prevalence of morbidity = 2%	Relative precision
Sub–Saharan Africa	DRC	20 000	1.8% to 2.2%	±10%
	Ghana	10 000	1.7% to 2.3%	±14%
	Kenya	20 000	1.8% to 2.2%	±10%
	Tanzania (2 sites)	15 000	1.8% to 2.2%	±11%
	Zambia	25 000	1.8% to 2.2%	±9%
	**Pooled**	**90 000**	**1.9% to 2.1%**	**±5%**
South Asia	Bangladesh	19 000	1.8% to 2.2%	±10%
	India	35000	1.9% to 2.1%	±7%
	Pakistan (2 sites)	16 000	1.8% to 2.2%	±11%
	**Pooled**	**70 000**	**1.9% to 2.1%**	**±5%**

CI – confidence interval, DRC – Democratic Republic of the Congo

### Data management

**Data processing.** The study uses paper forms or tablet–based software for data collection. Forms are independently double entered by two clerks into study databases with stringent range and consistency (R&C) checks with the exception of Zambia where field monitors collect data using forms designed in the TeleForms^®^ system (HP, Cambridge, UK). After Zambian supervisors review the forms for completeness, they are scanned, entered, and exported into an Access database. Similar R&C checks are built into the software used for data capture at sites using tablets. Data managers within the sites conduct inter–database checks to reconcile and synchronize data from various forms using the woman’s unique study ID as the link. Cleaned data are saved on special study servers with password–protected access to only principal investigators in the sites. They generate data back–ups on external drives at regular intervals. Every three months, sites transfer back–up data to a dedicated server at the WHO/MCA for external quality control and storage.

**Data analyses.** Analyses will be done using Stata^®^ statistical software package [24]. Incidence of severe maternal morbidities will be estimated. The burden of adverse birth outcomes will also be estimated. Associations will be independently explored between various maternal characteristics (confounders) such as socio–economic status, educational attainment, age, parity, etc. and severe maternal morbidity as well as the adverse birth outcomes. The effect of exposure to severe acute maternal morbidity on adverse birth outcomes will be estimated using appropriate regression models. Test of interaction will be done to assess effect modification of treatment received by study women on association between severe maternal morbidities and adverse pregnancy, birth and neonatal outcomes.

### Quality monitoring

The WHO/MCA centrally coordinates and monitors the harmonized implementation, quality of fieldwork and data in the AMANHI morbidity study. Individual sites send monthly fieldwork progress reports to WHO/MCA, highlighting their key challenges. At quarterly intervals, the WHO/MCA team run quality control checks on all transferred data to identify outliers and provide feedback to the sites. Data are also reconciled with the monthly fieldwork progress reports to check consistency. WHO/MCA sends experts to the sites once or twice each year to assess progress and quality of implementation, provide technical input and to enhance the harmonized implementation. They also discuss challenges with the sites and provide a detailed report to the WHO/MCA highlighting key issues of benefit to and for follow–up with the other sites.

### Ethical considerations

All women are individually consented to participate in the AMANHI morbidity study. Local and institutional ethics committees from all nine sites approved the AMANHI study protocols. The Ethics Review Committee of the WHO has also approved a combined master protocol with components on the role of the WHO/MCA.

### Dissemination plan

The results of the study will be disseminated among the public health, maternal and newborn health community of researchers, policy–makers and program managers. Channels for dissemination will include peer–reviewed journals, print and electronic media and academic presentations (oral and poster) at appropriate fora. In each participating country, there will be extensive briefing on their country–specific and overall study results, and the team of researchers and stakeholders will discuss implications of the study for interventions and programmes in those settings.

## Importance of the AMANHI morbidity study

Inadequate attention to reducing the burden of maternal morbidity may be contributing to the slow progress in reducing preventable maternal mortality [25]. Beyond survival, another significant statistic is the number of women who develop severe acute morbidities and/or severe chronic disabilities that are incompatible with normal physical, psychological or economic viability and who are abandoned by loved ones, families, friends and society [4,5,7]. One of the biggest hurdles to planning and delivery of effective interventions is the dearth of data on maternal morbidities.^11^ Good quality data are essential for strategic planning and targeting of interventions. In LMICs of sub–Saharan Africa and South Asia where resources are limited and a disproportionate burden of severe acute maternal morbidities exists, evidence–based data–driven strategic prioritization of investments and resource allocation to address these is paramount [4,6,26,27].

The AMANHI maternal morbidity study will generate reliable estimates of severe maternal morbidity from one of the largest population–based, multi–country studies in sub–Saharan Africa and South Asia. AMANHI has many advantages; implementation is being harmonized across sites and common definitions of severe maternal morbidity are being used. This will ensure comparability of data and facilitate pooled analyses across sites. The methodological contributions and implications of the AMANHI study design for routine data collection platforms such as demographic and health surveillance sites is obvious: the need for validation of definitions of morbidities and harmonization of protocols for data collection across sites is urgent. The absence of these limits data utility in routine surveillance systems especially where regional or global estimates are to be derived from these data. If AMANHI tools can be validated within other routine surveillance systems, it is a major contribution to harmonization of data collection tools or if not directly, provides the template for developing such valid, reliable and globally useful tool for population–based data collection systems.

The AMANHI sample size is large and with the active pregnancy and birth surveillance allowing for accurate denominators, estimates generated will be precise and reliable. The combined comparative advantages of large sample size and homogeneity in the data across sites will additionally allow for analyses on very rare maternal health outcomes and with the linked data on household wealth, inequities in the distribution of maternal morbidities could be explored.

AMANHI will provide the dual benefit of a unique opportunity to assess associations between various exposures, severe maternal morbidity and adverse pregnancy outcomes and also address the gap in the availability of quality data for validation of model–based estimates. The data will also form the baseline for generation of more accurate estimates of the real impact of severe acute morbidities on health and well–being of women after pregnancy and childbirth. Moreover, the implementation strategy informs global researchers, academics, funders and institutions on how to maximise the utility of data from on–going studies that could contribute to answering related questions.

While this contribution of reliable and good quality data on maternal morbidity from the AMANHI study to global public health is significant in that it will inform policy direction, interventions and programmes, we do recognize that it remains the first step needed to create a sustainable platform for prioritization and ensuring equitable coverage of maternal health interventions for the benefit of both mothers and their newborns.
