# Supplementary material for: Burden of severe maternal morbidity and association with adverse birth outcomes in sub–Saharan Africa and south Asia: protocol for a prospective cohort study
Source: J Glob Health. 2016 Sep 8;6(2):020601. doi: 10.7189/jogh.6.020601 (PMC5019012; doi:10.7189/jogh.6.020601)
Supplement: Online Supplementary Document [file jogh-06-020601-s001.pdf]

Online Supplementary Document

AMANHI Maternal Morbidity study group. Burden of severe maternal morbidity and association with adverse birth outcomes in sub–Saharan Africa and south Asia: protocol for a prospective cohort study

J Glob Health 2016;6:020601

OBJECTIVE 2 CORE VARIABLES BY FORM

ANTENATAL FORM (24-28 WEEKS, 32-36 WEEKS, 38-40 weeks)					
**No.**	**Question**	**Outcomes**	**Variable Name**	**Field Type**	**Size**
1	Date of filling the form<br>(all dates dd/mm/yyyy)	01/06/2010 to 30/04/2013<br>(sites to specify date range)	ADATEVISIT	Date	8
2	Number of visit	1=24-28 weeks gestation, 2=32-36 weeks gestation, 3= 38-40 weeks gestation	NUMANVISIT	Numeric	1
3	Study site	11=Bangladesh, 12=DRC, 13=Ghana, 15=India Shivgarh, 16=Kenya, 17=Pakistan Karachi, 18=Pakistan Matiari, 20=Tanzania Pemba, 21=Zambia	SITE	Numeric	2
4	Worker code	Each site to define valid range	FW	Text	3
5	Woman ID	Each site to define valid range	WHOWID	Text	10
6	Was the pregnant woman available for interview?	11=present, 12=currently in hospital, 13=temporarily absent, 14=died, 15=permanently moved out, 16=refused temporarily, 17=withdrawn consent, 18=visit could not be made	AWSTATUS	Numeric	2
	If visit could not be made, specify the reason	Text	NA_REASON	Text	50
7	If pregnant woman died, date of death	01/06/2010 to 30/04/2013, 08/08/1908=don't know, 09/09/1909=not applicable	AWDATEDIED	Date	8

ANTENATAL FORM (24-28 WEEKS, 32-36 WEEKS, 38-40 weeks)					
**No.**	**Question**	**Outcomes**	**Variable Name**	**Field Type**	**Size**
8	Are you still pregnant?	1=yes, 2=no, 8=don't know, 9=not applicable	APREGNANT	Numeric	1
9	If no, what was the outcome of your recently reported pregnancy?	11=miscarriage/abortion, 12= still birth, 13=live born baby alive, , 14=was not pregnant(false alarm/ previously falsely reported), 15=still pregnant, 88= don't know, 99= not applicable (woman died)	AENDPREG	Numeric	2
10	If no longer pregnant, when did the recent pregnancy end?	01/06/2010 to 30/04/2013 (sites to specify date range), 08/08/1908=don't know, 09/09/1909=NA	ADATEDEL	Date	8
11	Which weeks of pregnancy is this?	Week 01 to 42, 88=don't know, 99=NA	WKPRE	Numeric	2
12	Which months of pregnancy is this?	Month 01 to 10, 88=don't know, 99=NA	MTHPREG	Numeric	2
**General Health**					
13	How would you rate your health today?	11=excellent, 12=moderate, 13=poor	AGHEALTH	Numeric	2
**Hemorrhage**					
14	During this pregnancy / since the last visit, were you told that you had a hemorrhage?	1=yes, 2=no, 8=don't know	ATOLDHEMO	Numeric	1
15	During this pregnancy / since the last visit, have you had any bleeding from the vagina?	1=yes, 2=no, 8=don't know	ABLEED	Numeric	1
16	Did the bleeding last for longer than one day?	1=yes, 2=no, 8=don't know, 9=NA	ALBLEED	Numeric	1
17	Did the bleeding wet your clothes, the bed or the floor?	1=yes, 2=no, 8=don't know, 9=NA	ASEVBLEED	Numeric	1
18	Was the blood fresh (bright red) compared to dark red and viscous?	1=fresh, 2=not fresh, 8=don't know, 9=NA	AFRESHBLD	Numeric	1

ANTENATAL FORM (24-28 WEEKS, 32-36 WEEKS, 38-40 weeks)					
**No.**	**Question**	**Outcomes**	**Variable Name**	**Field Type**	**Size**
19	Did you lose consciousness around the time of or because of the bleeding?	1=yes, 2=no, 8=don't know, 9=NA	ABLDCON	Numeric	1
**Hypertensive Disorders of Pregnancy**					
20	During this pregnancy / since the last visit, were you told that you have high blood pressure?	1=yes, 2=no, 8=don't know	ABPTOLD	Numeric	1
21	Measure systolic blood pressure and record results	xxx-yyy, 888=don't know, 999=NA	ABPSYS	Numeric	3
22	Measure diastolic blood pressure and record results	xxx-yyy, 888=don't know, 999=NA	ABPDIAS	Numeric	3
23	Measure proteinuria using dipstick and record result	1=2+, 2=1+, 3=normal, 8=don't know, 9=NA	ADIPSTICK	Numeric	1
24	During this pregnancy / since the last visit, did you have a headache?	1=yes, 2=no, 8=don't know	AHEAD	Numeric	1
25	For how long did you have a headache?	xx-yy days, 88=don't know, 99=NA	ALHEAD	Numeric	2
26	Was the headache severe?	1=yes, 2=no, 8=don't know, 9=NA	ASEVHEAD	Numeric	1
27	During this pregnancy / since the last visit, did you have any swelling?	1=yes, 2=no, 8=don't know	ASWELL	Numeric	1
28	For how long did you have the swelling?	xx-yy days, 88=don't know, 99=NA	ALSWELL	Numeric	2
29	Was the swelling on your whole body?	1=yes, 2=no, 8=don't know, 9=NA	ABDYSWELL	Numeric	1
30	Was the swelling on your joints?	1=yes, 2=no, 8=don't know, 9=NA	AJNTSWELL	Numeric	1
31	Was the swelling on your ankles?	1=yes, 2=no, 8=don't know, 9=NA	AANKSWELL	Numeric	1
32	Was the swelling on your face?	1=yes, 2=no, 8=don't know, 9=NA	AFACSWELL	Numeric	1

ANTENATAL FORM (24-28 WEEKS, 32-36 WEEKS, 38-40 weeks)					
**No.**	**Question**	**Outcomes**	**Variable Name**	**Field Type**	**Size**
	(Did you have a puffy face?)				
33	If the swelling was on any other part of the body, please specify where	Text	AOTHSWELL	Text	15
34	During this pregnancy / since the last visit, did you have blurred vision?	1=yes, 2=no, 8=don't know	AVISION	Numeric	1
35	For how long did you have blurred vision?	xx-yy days, 88=don't know, 99=NA	ALVISION	Numeric	2
36	During this pregnancy / since the last visit have you had convulsions?	1=yes, 2=no, 8=don't know	ACONVULSE	Numeric	1
37	For how long did you have convulsions?	xx-yy days, 88=don't know, 99=NA	ALCONVULS	Numeric	1
38	Did you lose consciousness because of the convulsions?	1=yes, 2=no, 8=don't know, 9=NA	ACONVCON	Numeric	1
**Fistula**					
39	During this pregnancy / since the last visit, did you experience continuously dripping urine?	1=yes, 2=no, 8=don't know	AURINEDRIP	Numeric	1
40	During this pregnancy / since the last visit, did you ever lose urine during sudden physical exertion, lifting, coughing or sneezing?	1=yes, 2=no, 8=don't know	AURINELOSE	Numeric	1
41	During this pregnancy / since the last visit, did you ever experience such a strong and sudden urge to urinate that you leak before reaching the toilet?	1=yes, 2=no, 8=don't know	AURINELEAK	Numeric	1
42	During this pregnancy / since the last visit, have you experienced	1=yes, 2=no, 8=don't know	AFECESLEAK	Numeric	1

ANTENATAL FORM (24-28 WEEKS, 32-36 WEEKS, 38-40 weeks)					
**No.**	**Question**	**Outcomes**	**Variable Name**	**Field Type**	**Size**
	feces passing through the birth canal?				
43	Has any physician or healthcare provider ever told you that you have a medical problem involving your vagina/pelvis or bladder while not pregnant?	1=yes, 2=no, 8=don't know	AVAGPROB	Numeric	1
44	Have you ever undergone surgery in the vagina/pelvis or bladder not including cesarean section, abortion, or surgery for contraception?	1=yes, 2=no, 8=don't know	AVAGSURG	Numeric	1
**Care Seeking**					
45	Were you in a facility when any of these symptoms occurred?	1=yes, 2=no, 8=don't know	AFACSYMP	Numeric	1
46	If you were at a facility when you experienced any of these symptoms, what type of facility was it?	11=government hospital, 12=government clinic/health centre, 13=private hospital, 14=private clinic/health centre, 66=other, 88=don't know, 99=NA	AFCTYPSYM	Numeric	2
47	If you were at a facility when you experienced any of these symptoms, were you referred to another facility?	1=yes, 2=no, 8=don't know, 9=NA	AFACREF	Numeric	1
48	If you were at a facility when you experienced any of these symptoms and were referred, what type of facility was it?	11=government hospital, 12=government clinic/health centre, 13=private hospital, 14=private clinic/health centre, 66=other, 88=don't know, 99=NA	AFCREFTYP	Numeric	2
49	If you were at a facility when you experienced any of these symptoms and were referred, did you go to the referral facility?	1=yes, 2=no, 8=don't know, 9=NA	AFACREFGO	Numeric	1

ANTENATAL FORM (24-28 WEEKS, 32-36 WEEKS, 38-40 weeks)					
**No.**	**Question**	**Outcomes**	**Variable Name**	**Field Type**	**Size**
50	If you were not at a facility when you experience any of these symptoms, did you seek care?	1=yes, 2=no, 9=NA	ASYMPSEEK	Numeric	1
51	If you sought care for any of the symptoms, for which symptoms did you seek care?	Bleeding: 1=yes, 2=no, 9=NA	ABLDSEEK	Numeric	1
		High blood pressure: 1=yes, 2=no, 9=NA	AHBPSEEK	Numeric	1
		Severe headache: 1=yes, 2=no, 9=NA	AHEADSEEK	Numeric	1
		Swelling: 1=yes, 2=no, 9=NA	ASWELSEEK	Numeric	1
		Blurred vision: 1=yes, 2=no, 9=NA	AVISSEEK	Numeric	1
		Convulsions: 1=yes, 2=no, 9=NA	ACONVSEEK	Numeric	1
		Loss of consciousness: 1=yes, 2=no, 9=NA	ALCSEEK	Numeric	1
		Leakage of urine or feces: 1=yes, 2=no, 9=NA	ALEAKSEEK	Numeric	1
52	If you sought care, what was the first place where you sought care?	11=government hospital, 12=government clinic/health centre, 13=private hospital, 14=private clinic/health centre, 15=community health worker, 16=traditional healer, 17=pharmacy/druggist,18=traditional birth attendant, 66=other, 99=NA	APLCCSEEK	Numeric	2
53	Where you admitted to sleep at a facility because of the symptoms you experienced?	1=yes, 2=no, 9=NA	AADMIN	Numeric	1
54	For how many nights were you admitted?	11=one day only, 12=1-3 days total, 13=4-6 days total, 14=more than 6 days total, 88=don't know, 99=NA	ANUMADMIN	Numeric	2
55	If you sought care, did you receive any of the following treatments?	Hysterectomy (operation to remove your womb): 1=yes, 2=no, 9=NA	AHYSTEREC	Numeric	1
		Laparotomy or other surgery (any operation where they put you to sleep and operated on your abdomen): 1=yes, 2=no, 9=NA	ASURGERY	Numeric	1

ANTENATAL FORM (24-28 WEEKS, 32-36 WEEKS, 38-40 weeks)					
**No.**	**Question**	**Outcomes**	**Variable Name**	**Field Type**	**Size**
		Blood transfusion: 1=yes, 2=no, 9=NA	ABLDTRANS	Numeric	1
		Assisted breathing (by hand or by machine): 1=yes, 2=no, 9=NA	AVENTILATE	Numeric	1
		IV fluid: 1=yes, 2=no, 9=NA	AIVFLUID	Numeric	1
		Drugs/pills/medications: 1=yes, 2=no, 9=NA	ADRUGS	Numeric	1
		Other treatment (specify)	AOTHTRTMT	Text	30
**MCH Document / ANC Card**					
56	Weight of woman (kg)	xx.x-yy.y, 99.9=NA	AFCWEIGHT	Numeric	3
57	Height of woman (cm)	xxx-yyy, 999=NA	AFCHEIGHT	Numeric	3
58	LMP	01/08/2009 to 30/04/2013 (sites to specify date range), 08/08/1908=don't know, 09/09/1909=NA	AFCLMP	Date	8
59	Number of antenatal visits	xx-yy, 99=NA	AFCNUMANC	Numeric	2
60	Estimated date of delivery	01/08/2009 to 30/04/2013 (sites to specify date range), 08/08/1908=don't know, 09/09/1909=NA	AFCDATDELIV	Date	8
61	Number of Admissions	xx-yy, 99=NA	FCNUMADM	Numeric	2
62	Treatments	Hysterectomy: 1=yes, 2=no, 9=NA	AFCHYSTER	Numeric	1
		Laparotomy or other surgery: 1=yes, 2=no, 9=NA	AFCSRGY	Numeric	1
		Blood transfusion: 1=yes, 2=no, 9=NA	AFCBLDTRN	Numeric	1
		Assisted breathing: 1=yes, 2=no, 9=NA	AFCVENTIL	Numeric	1
		IV fluid: 1=yes, 2=no, 9=NA	AFCIVFLUID	Numeric	1
		Drugs/pills/medications: 1=yes, 2=no, 9=NA	AFCDRUGS	Numeric	1
		Other treatment (specify)	AFCOTHER	Text	30

POSTNATAL 1 FORM (1-6 days after delivery)					
**No.**	**Question**	**Outcomes**	**Variable Name**	**Field Type**	**Size**
1	Date of filling the form<br>(all dates dd/mm/yyyy)	01/06/2010 to 30/04/2013<br>(sites to specify date range)	P1DATEVISIT	Date	8
2	Study site	11=Bangladesh, 12=DRC, 13=Ghana, 15=India Shivgarh, 16=Kenya, 17=Pakistan Karachi, 18=Pakistan Matiari, 20=Tanzania Pemba, 21=Zambia	SITE	Numeric	2
3	Worker code	Each site to define valid range	FW	Text	3
4	Woman ID	Each site to define valid range	WHOWID	Text	10
5	Did the mother deliver more than one child	1=yes, 2=no	MULTI	Numeric	1
6	If yes, how many?	xx, 99=NA	NMULTI	Numeric	1
7	How many of these were live born?	Xx, 99=NA	LIVEMULTI	Numeric	1
8	Baby ID (only for live births)	Each site to define valid range, 99=NA	WHOBID	Text	10
8.1	Baby ID 2 (only for live births)<br><br>Continue with 8.2 and WHOBID3 etc. if needed	Each site to define valid range, 99=NA	WHOBID2	Text	10
9	Live births/stillbirth:<br>Date of birth<br><br>Miscarriage/abortion:<br>Date of miscarriage/abortion	Each site to define valid range	P1DATEB	Date	8
10	Was the mother available for interview?	11=present, 12=currently in hospital, 13=temporarily absent, 14=died, 15=permanently moved out, 16=refused temporarily, 17=withdrawn consent, 18=visit could not be made	P1WSTATUS	Numeric	2

POSTNATAL 1 FORM (1-6 days after delivery)					
**No.**	**Question**	**Outcomes**	**Variable Name**	**Field Type**	**Size**
11	If visit could not be made, specify the reason	Text	P1M_REASON	Text	50
12	If mother died, date of death	01/06/2010 to 30/04/2013 (sites to specify date range), 08/08/1908=don't know, 09/09/1909=NA	MDATEDIED	Date	8
13	Baby's status at time of interview	Live births:<br>11=present, 12=currently in hospital, 13=temporarily absent, 14=died, 15=permanently moved out, 16=refused temporarily, 17=withdrawn consent, 18=visit could not be made<br><br>Other:<br>88=miscarriage/abortion, 99=baby stillborn	P1BSTATUS	Numeric	2
13.1	Baby 2's status at time of interview<br><br>Continue with 13.2 and P1BSTATUS3 etc. if needed	Live births:<br>11=present, 12=currently in hospital, 13=temporarily absent, 14=died, 15=permanently moved out, 16=refused temporarily, 17=withdrawn consent, 18=visit could not be made<br><br>Other:<br>88=miscarriage/abortion, 99=baby stillborn	P1BSTATUS2		
14	If visit could not be made, specify the reason	Text	P1B_REASON	Text	50
14.1	If visit could not be made for baby 2, specify the reason<br><br>Continue with 14.2 and P1B_REASON3 etc. if needed	Text	P1B_REASON2	Text	50

POSTNATAL 1 FORM (1-6 days after delivery)					
**No.**	**Question**	**Outcomes**	**Variable Name**	**Field Type**	**Size**
15	If baby died, date of death<br>(not applicable for miscarriage/abortion)	01/06/2010 to 30/04/2013 (sites to specify date range), 08/08/1908=don't know, 09/09/1909=NA	BDATEDIED	Date	8
15.1	If baby 2 died, date of death<br><br>Continue with 15.2 and BDATEDIED3 etc. if needed	01/06/2010 to 30/04/2013 (sites to specify date range), 08/08/1908=don't know, 09/09/1909=NA	BDATEDIED2	Date	8
16	How many hours after birth did you first put the baby to the breast?<br>(not applicable for miscarriage/abortion)	000 to 096, 888 = don't know, 999= not applicable	P1BFSTART	Numeric	3
16.1	How many hours after birth did you first put baby 2 to the breast?<br><br>Continue with 16.2 and P1BFSTART3 etc. if needed	000 to 096, 888 = don't know, 999= not applicable	P1BFSTART2	Numeric	3
**General Health**					
17	How would you rate your health today?	11=excellent, 12=moderate, 13=poor	P1GHEALTH	Numeric	2
**Labor (only for live births or stillbirths)**					
18	When did your water break?	1=before labor started, 2=after labor started or during delivery, 8=don't know, 9=NA	P1WTRBRK	Numeric	1
19	How much time before delivery did the waters break?	1=<24 hours, 2=24 hours or more, 8=don't know, 9=NA (broke during delivery/did not break before C-section)	P1WTRBRKT	Numeric	1
20	Were the waters clear?	1=clear, 2=not clear/dark, 8=don't know, 9=NA (waters didn't break)	P1WTRCLR	Numeric	1

POSTNATAL 1 FORM (1-6 days after delivery)					
**No.**	**Question**	**Outcomes**	**Variable Name**	**Field Type**	**Size**
21	How much time before delivery did your labor pains start?	1=<24 hours, 2=24 hours or more, 8=don't know, 9=NA	P1LBRSTART	Numeric	1
**Delivery (only for live births or stillbirths)**					
22	Where did the delivery take place?	1=hospital, 2=other health facility, 3=home, 6=other (specify), 8=don't know, 9=NA	P1PLCDELIV	Numeric	1
23	Who assisted the delivery	1=doctor, 2=nurse/midwife, 3=TBA, 4=relative, 5=mother herself, 6=other (specify), 8=don't know, 9=NA	P1ATNDNT	Numeric	1
24	What type of delivery was it?	1=normal vaginal, 2=assisted vaginal (forceps/vacuum), 3=Cesarean section, 8=don't know, 9=NA	P1TYPDELIV	Numeric	1
25	Which part of the baby came out first?	1=head, 2=bottom, 3=feet, 4=arm/hand, 6=other (specify), 8=don't know, 9=NA (C/S)	P1PRTDELIV	Numeric	1
25.1	For baby 2:<br>Which part of the baby came out first<br><br>Continue with 25.2 and P1PRTDELIV3 etc. if needed	1=head, 2=bottom, 3=feet, 4=arm/hand, 6=other (specify), 8=don't know, 9=NA (C/S)	P1PRTDELIV2	Numeric	1
26	If vaginal delivery, did the umbilical cord come out before the baby?	1=yes, 2=no, 8=don't know, 9=NA	P1CORD	Numeric	1
26.1	For baby 2:<br>If vaginal delivery, did the umbilical cord come out before the baby?<br><br>Continue with 26.2 and P1CORD3 etc. if needed	1=yes, 2=no, 8=don't know, 9=NA	P1CORD2	Numeric	1

POSTNATAL 1 FORM (1-6 days after delivery)					
**No.**	**Question**	**Outcomes**	**Variable Name**	**Field Type**	**Size**
27	Where was the cord when the baby came out?	1=not around the baby, 2=around the neck, 3=around the body, 4=around other (specify), 8=don’t know, 9=NA	P1CORDPLC	Numeric	1
27.1	For baby 2:<br>Where was the cord when the baby came out?<br><br>Continue with 27.2 and P1CORDPLC3 etc. if needed	1=not around the baby, 2=around the neck, 3=around the body, 4=around other (specify), 8=don’t know, 9=NA	P1CORDPLC2	Numeric	1
28	Did the baby cry, move or breathe after birth?	1=yes, 2=no, 8=don’t know, 9=NA	P1STILLB	Numeric	1
28.1	For baby 2:<br>Did the baby cry, move or breathe after birth?<br><br>Continue with 28.2 and P1STILLB3 etc. if needed	1=yes, 2=no, 8=don’t know, 9=NA	P1STILLB2	Numeric	1
29	If you had a cesarean section, did you know before you went into labor that you would have a cesarean section?	1=yes, 2=no, 8=don’t know, 9=NA	P1KNOWCS	Numeric	1
30	If you had a cesarean section, is there pus coming from the scar?	1=yes, 2=no, 8=don’t know, 9=NA	P1PUSCS	Numeric	1
31	If you had a cesarean section, was it because the baby was too big?	1=yes, 2=no, 8=don’t know, 9=NA	P1CSBBIG	Numeric	1
32	If you had a cesarean section, was it because the baby was lying in a bad position?	1=yes, 2=no, 8=don’t know, 9=NA	P1CSBPOS	Numeric	1

POSTNATAL 1 FORM (1-6 days after delivery)					
**No.**	**Question**	**Outcomes**	**Variable Name**	**Field Type**	**Size**
33	If you had a cesarean section, was it because you had a small pelvis?	1=yes, 2=no, 8=don't know, 9=NA	P1CSSMALL	Numeric	1
34	If you had a cesarean section, was it because you had a ruptured uterus or your uterus might rupture?	1=yes, 2=no, 8=don't know, 9=NA	P1CSURUPT	Numeric	1
35	If you had a cesarean section, was it because your uterus was too weak?	1=yes, 2=no, 8=don't know, 9=NA	P1CSUWEAK	Numeric	1
36	If you had a cesarean section, was it because of bleeding?	1=yes, 2=no, 8=don't know, 9=NA	P1CSBLEED	Numeric	1
37	If you had a cesarean section, was it because of convulsions?	1=yes, 2=no, 8=don't know, 9=NA	P1CSCONV	Numeric	1
38	Did the placenta come out within one hour of delivery?	1=yes, 2=no, 8=don't know, 9=NA	P1PLACENTA	Numeric	1
39	Did you have an episiotomy or did anyone cut your vagina during delivery?	1=yes, 2=no, 8=don't know, 9=NA	P1EPISIOT	Numeric	1
40	If yes, is there pus coming from the cut/tear?	1=yes, 2=no, 8=don't know, 9=NA	P1INFECEPIS	Numeric	1
**Hemorrhage (applicable for all)**					
41	Since the last visit, were you told that you had a hemorrhage?	1=yes, 2=no, 8=don't know	P1TLDHEMO	Numeric	1
42	Live birth/stillbirth:<br>If yes, were you told you had a hemorrhage before delivery?<br><br>Miscarriage/abortion:<br>If yes, were you told you had a	1=yes, 2=no, 8=don't know, 9=NA	P1TLDHEMBD	Numeric	1

POSTNATAL 1 FORM (1-6 days after delivery)					
**No.**	**Question**	**Outcomes**	**Variable Name**	**Field Type**	**Size**
	hemorrhage before the miscarriage/abortion?				
43	Live birth/stillbirth:<br>If yes, were you told you had a hemorrhage during delivery?<br><br>Miscarriage/abortion:<br>If yes, were you told you had a hemorrhage during the miscarriage/ abortion?	1=yes, 2=no, 8=don't know, 9=NA	P1TLDHEMD	Numeric	1
44	Live birth/stillbirth:<br>If yes, were you told you had a hemorrhage after delivery?<br><br>Miscarriage/abortion:<br>If yes, were you told you had a hemorrhage after the miscarriage/ abortion?	1=yes, 2=no, 8=don't know, 9=NA	P1TLDHEMAD	Numeric	1
45	Since the last visit, have you had any bleeding from the vagina?	1=yes, 2=no, 8=don't know	P1BLEED	Numeric	1
46	Live birth/stillbirth:<br>If yes, did the bleeding from the vagina occur before delivery?<br><br>Miscarriage/abortion:<br>If yes, did the bleeding from the vagina occur before the miscarriage/ abortion?	1=yes, 2=no, 8=don't know, 9=NA	P1BLEEDBD	Numeric	1
47	Live birth/stillbirth:<br>If yes, did the bleeding from the vagina occur during delivery?	1=yes, 2=no, 8=don't know, 9=NA	P1BLEEDD	Numeric	1

POSTNATAL 1 FORM (1-6 days after delivery)					
**No.**	**Question**	**Outcomes**	**Variable Name**	**Field Type**	**Size**
	Miscarriage/abortion<br>If yes, did the bleeding from the vagina occur during the miscarriage/ abortion?				
48	Live birth/stillbirth:<br>If yes, did the bleeding from the vagina occur after delivery?<br><br>Miscarriage/abortion:<br>If yes, did the bleeding from the vagina occur after the miscarriage/ abortion?	1=yes, 2=no, 8=don't know, 9=NA	P1BLEEDAD	Numeric	1
49	Did the bleeding last for longer than one day?	1=yes, 2=no, 8=don't know, 9=NA	P1LBLEED	Numeric	1
50	Did the bleeding wet your clothes, the bed or the floor?	1=yes, 2=no, 8=don't know, 9=NA	P1SEVBLEED	Numeric	1
51	Was the blood fresh (bright red) compared to dark red and viscous?	1=fresh, 2=not fresh, 8=don't know, 9=NA	P1FRESHBLD	Numeric	1
52	Did you lose consciousness around the time of or because of the bleeding?	1=yes, 2=no, 8=don't know, 9=NA	P1BLDCON	Numeric	1
**Hypertensive Disorders of Pregnancy (applicable for all)**					
53	Since the last visit, were you told that you have high blood pressure?	1=yes, 2=no, 8=don't know	P1BPTOLD	Numeric	1
54	Measure systolic blood pressure and record results	xxx-yyy, 888=don't know, 999=NA	P1BPSYS	Numeric	3
55	Measure diastolic blood pressure and record results	xxx-yyy, 888=don't know, 999=NA	P1BPDIAS	Numeric	3

POSTNATAL 1 FORM (1-6 days after delivery)					
**No.**	**Question**	**Outcomes**	**Variable Name**	**Field Type**	**Size**
56	Measure proteinuria using dipstick and record result	1=2+, 2=1+, 3=normal, 8=don't know, 9=NA	P1DIPSTICK	Numeric	1
57	Since the last visit, did you have a headache?	1=yes, 2=no, 8=don't know	P1HEAD		
58	For how long did you have a headache?	xx-yy days, 88=don't know, 99=NA	P1LHEAD	Numeric	2
59	Was the headache severe?	1=yes, 2=no, 8=don't know, 9=NA	P1SEVHEAD	Numeric	1
60	Live birth/stillbirth:<br>If yes, did the severe headache occur before delivery?<br><br>Miscarriage/abortion:<br>If yes, did the severe headache occur before the miscarriage/ abortion?	1=yes, 2=no, 8=don't know, 9=NA	P1SHEADBD	Numeric	1
61	Live birth/stillbirth:<br>If yes, did the severe headache occur during delivery?<br><br>Miscarriage/abortion:<br>If yes, did the severe headache occur during the miscarriage/ abortion?	1=yes, 2=no, 8=don't know, 9=NA	P1SHEADD	Numeric	1
62	Live birth/stillbirth:<br>If yes, did the severe headache occur after delivery?<br><br>Miscarriage/abortion:<br>If yes, did the severe headache occur after the miscarriage/	1=yes, 2=no, 8=don't know, 9=NA	P1SHEADAD	Numeric	1

POSTNATAL 1 FORM (1-6 days after delivery)					
**No.**	**Question**	**Outcomes**	**Variable Name**	**Field Type**	**Size**
	abortion?				
63	Since the last visit, did you have any swelling?	1=yes, 2=no, 8=don't know	P1SWELL	Numeric	1
64	Live birth/stillbirth:<br>If yes, did you have swelling before delivery?<br><br>Miscarriage/abortion:<br>If yes, did you have swelling before the miscarriage/ abortion?	1=yes, 2=no, 8=don't know, 9=NA	P1SWELLBD	Numeric	1
65	Live birth/stillbirth:<br>If yes did you have swelling during delivery?<br><br>Miscarriage/abortion:<br>If yes did you have swelling during the miscarriage/ abortion?	1=yes, 2=no, 8=don't know, 9=NA	P1SWELLD	Numeric	1
66	Live birth/stillbirth:<br>If yes, did you have swelling after delivery?<br><br>Miscarriage/abortion:<br>If yes, did you have swelling after the miscarriage/ abortion?	1=yes, 2=no, 8=don't know, 9=NA	P1SWELLAD	Numeric	1
67	For how long did you have the swelling?	xx-yy days, 88=don't know, 99=NA	P1LSWELL	Numeric	2
68	Was the swelling on your whole body?	1=yes, 2=no, 8=don't know, 9=NA	P1BDYSWELL	Numeric	1

POSTNATAL 1 FORM (1-6 days after delivery)					
**No.**	**Question**	**Outcomes**	**Variable Name**	**Field Type**	**Size**
69	Was the swelling on your joints?	1=yes, 2=no, 8=don't know, 9=NA	P1JNTSWELL	Numeric	1
70	Was the swelling on your ankles?	1=yes, 2=no, 8=don't know, 9=NA	P1ANKSWELL	Numeric	1
71	Was the swelling on your face? (Did you have a puffy face?)	1=yes, 2=no, 8=don't know, 9=NA	P1FACSWELL	Numeric	1
72	If the swelling was on any other part of the body, please specify where	Text	P1OTHSWELL	Text	15
73	Since the last visit, did you have blurred vision?	1=yes, 2=no, 8=don't know	P1VISION	Numeric	1
74	Live birth/stillbirth:<br>If yes, did you have blurred vision before delivery?<br><br>Miscarriage/abortion:<br>If yes, did you have blurred vision before the miscarriage/ abortion?	1=yes, 2=no, 8=don't know , 9=NA	P1VISIONBD	Numeric	1
75	Live birth/stillbirth:<br>If yes, did you have blurred vision during delivery?<br><br>Miscarriage/abortion:<br>If yes, did you have blurred vision during the miscarriage/ abortion?	1=yes, 2=no, 8=don't know , 9=NA	P1VISIOND	Numeric	1

POSTNATAL 1 FORM (1-6 days after delivery)					
**No.**	**Question**	**Outcomes**	**Variable Name**	**Field Type**	**Size**
76	Live birth/stillbirth:<br>If yes, did you have blurred vision after delivery?<br><br>Miscarriage/abortion:<br>If yes, did you have blurred vision after the miscarriage/ abortion?	1=yes, 2=no, 8=don't know , 9=NA	P1VISIONAD	Numeric	1
77	For how long did you have blurred vision?	xx-yy days, 88=don't know, 99=NA	P1LVISION	Numeric	2
78	Since the last visit have you had convulsions?	1=yes, 2=no, 8=don't know	P1CONVULSE	Numeric	1
79	Live birth/stillbirth:<br>If yes, did you have convulsions before delivery?<br><br>Miscarriage/abortion:<br>If yes, did you have convulsions before the miscarriage/ abortion?	1=yes, 2=no, 8=don't know , 9=NA	P1CONVSBD	Numeric	1
80	Live birth/stillbirth:<br>If yes, did you have convulsions during delivery?<br><br>Miscarriage/abortion:<br>If yes, did you have convulsions during the miscarriage/ abortion?	1=yes, 2=no, 8=don't know , 9=NA	P1CONVD	Numeric	1
81	Live birth/stillbirth:<br>If yes, did you have convulsions	1=yes, 2=no, 8=don't know , 9=NA	P1CONVAD	Numeric	1

POSTNATAL 1 FORM (1-6 days after delivery)					
**No.**	**Question**	**Outcomes**	**Variable Name**	**Field Type**	**Size**
	after delivery?<br><br>Miscarriage/abortion:<br>If yes, did you have convulsions after the miscarriage/ abortion?				
82	For how long did you have convulsions?	xx-yy days, 88=don't know, 99=NA	P1LCONV	Numeric	1
83	Did you lose consciousness because of the convulsions?	1=yes, 2=no, 8=don't know, 9=NA	P1CONVCON	Numeric	1
**Sepsis (applicable for all)**					
84	Since my last visit, have you had a fever?	1=yes, 2=no, 8=don't know	P1FEVER	Numeric	1
85	Live birth/stillbirth:<br>If yes, did the fever occur before delivery?<br><br>Miscarriage/abortion:<br>If yes, did the fever occur before the miscarriage/ abortion?	1=yes, 2=no, 8=don't know, 9=NA	P1FEVERBD	Numeric	1
86	Live birth/stillbirth:<br>If yes, did the fever occur during delivery?<br><br>Miscarriage/abortion:<br>If yes, did the fever occur during the miscarriage/ abortion?	1=yes, 2=no, 8=don't know, 9=NA	P1FEVERD	Numeric	1
87	Live birth/stillbirth:<br>If yes, did the fever occur after delivery?	1=yes, 2=no, 8=don't know, 9=NA	P1FEVERAD	Numeric	1

POSTNATAL 1 FORM (1-6 days after delivery)					
**No.**	**Question**	**Outcomes**	**Variable Name**	**Field Type**	**Size**
	Miscarriage/abortion:<br>If yes, did the fever occur after the miscarriage/ abortion?				
88	If yes, did the fever last more than one day?	1=yes, 2=no, 8=don't know, 9=NA	P1LFEVER	Numeric	1
89	Since my last visit, did you have smelly discharge or pus pass from your vagina?	1=yes, 2=no, 8=don't know	P1VDISCHG	Numeric	1
90	If yes, when did the smelly discharge or pus pass from your vagina?	1=before delivery, 2=after delivery, 8=don't know, 9=NA	P1VDISCHGT	Numeric	1
91	Since the last visit, were you told that you have sepsis?	1=yes, 2=no, 8=don't know	P1SEPSIS	Numeric	1
92	If yes, when were you told that you have sepsis?	1=before delivery, 2=after delivery, 8=don't know, 9=NA	P1SEPSISTIM	Numeric	1
**Fistula (applicable for all)**					
93	Since the last visit, did you experience continuously dripping urine?	1=yes, 2=no, 8=don't know	P1URINDRIP	Numeric	1
94	Since the last visit, did you ever lose urine during sudden physical exertion, lifting, coughing or sneezing?	1=yes, 2=no, 8=don't know	P1URINLOSE	Numeric	1
95	Since the last visit, did you ever experience such a strong and sudden urge to urinate that you leak before reaching the toilet?	1=yes, 2=no, 8=don't know	P1URINLEAK	Numeric	1
96	Since the last visit, have you experienced feces passing through the birth canal?	1=yes, 2=no, 8=don't know	P1FECLEAK	Numeric	1

POSTNATAL 1 FORM (1-6 days after delivery)					
**No.**	**Question**	**Outcomes**	**Variable Name**	**Field Type**	**Size**
97	Since the last visit, has any physician or healthcare provider told you that you have a medical problem involving your vagina/pelvis or bladder while not pregnant?	1=yes, 2=no, 8=don't know	P1VAGPROB	Numeric	1
98	Since the last visit, have you undergone surgery in the vagina/pelvis or bladder not including cesarean section, abortion, or surgery for contraception?	1=yes, 2=no, 8=don't know	P1VAGSURG	Numeric	1
**Care Seeking (applicable for all)**					
99	Were you in a facility when any of these symptoms occurred?	1=yes, 2=no, 8=don't know, 9=NA	P1FSYMP	Numeric	1
100	If you were at a facility when you experienced any of these symptoms, what type of facility was it?	11=government hospital, 12=government clinic/health centre, 13=private hospital, 14=private clinic/health centre, 66=other, 88=don't know, 99=NA	P1FTYPSYM	Numeric	2
101	If you were at a facility when you experienced any of these symptoms, were you referred to another facility?	1=yes, 2=no, 8=don't know, 9=NA	P1FREF	Numeric	1
102	If you were at a facility when you experienced any of these symptoms and were referred, what type of facility was it?	11=government hospital, 12=government clinic/health centre, 13=private hospital, 14=private clinic/health centre, 66=other, 88=don't know, 99=NA	P1FREFTYP	Numeric	2
103	If you were at a facility when you experienced any of these symptoms and were referred, did you go to the referral	1=yes, 2=no, 8=don't know, 9=NA	P1FREFGO	Numeric	1

POSTNATAL 1 FORM (1-6 days after delivery)					
**No.**	**Question**	**Outcomes**	**Variable Name**	**Field Type**	**Size**
	facility?				
104	If you were not at a facility when you experience any of these symptoms, did you seek care?	1=yes, 2=no, 9=NA	P1SYMPSK	Numeric	1
	If you sought care for any of the symptoms, for which symptoms did you seek care?	Bleeding: 1=yes, 2=no, 9=NA	P1BLDSK	Numeric	1
		High blood pressure: 1=yes, 2=no, 9=NA	P1HBPSK	Numeric	1
		Severe headache: 1=yes, 2=no, 9=NA	P1HEADSK	Numeric	1
		Swelling: 1=yes, 2=no, 9=NA	P1SWELSK	Numeric	1
		Blurred vision: 1=yes, 2=no, 9=NA	P1VSNSK	Numeric	1
		Convulsions: 1=yes, 2=no, 9=NA	P1CONVSK	Numeric	1
		Loss of consciousness: 1=yes, 2=no, 9=NA	P1LCSK	Numeric	1
		Fever: 1=yes, 2=no, 9=NA	P1FVRSK	Numeric	1
		Vaginal discharge/pus: 1=yes, 2=no, 9=NA	P1VAGDISK	Numeric	1
		Leakage of urine or feces: 1=yes, 2=no, 9=NA	P1LEAKSK	Numeric	1
105	If you sought care, what was the first place where you sought care??	11=government hospital, 12=government clinic/health centre, 13=private hospital, 14=private clinic/health centre, 15=community health worker, 16=traditional healer, 17=pharmacy/druggist, 18=traditional birth attendant, 66=other, 99=NA	P1PLCCSK	Numeric	2
106	Where you admitted to sleep at a facility because of the symptoms you experienced?	1=yes, 2=no, 9=NA	P1ADMIN	Numeric	1
107	For how many nights were you admitted?	11=one day only, 12=1-3 days total, 13=4-6 days total, 14=more than 6 days total, 88=don’t know, 99=NA	P1NUMADM	Numeric	2
108	If you sought care, did you receive any of the following	Hysterectomy (operation to remove your womb): 1=yes, 2=no, 9=NA	P1HYSTER	Numeric	1

POSTNATAL 1 FORM (1-6 days after delivery)					
**No.**	**Question**	**Outcomes**	**Variable Name**	**Field Type**	**Size**
	treatments?	Laparotomy or other surgery (any operation where they put you to sleep and operated on your abdomen): 1=yes, 2=no, 9=NA	P1SURGERY	Numeric	1
		Tear in vagina repaired: 1=yes, 2=no, 9=NA	P1REPTEAR	Numeric	1
		Blood transfusion: 1=yes, 2=no, 9=NA	P1BLDTRANS	Numeric	1
		Assisted breathing (by hand or by machine): 1=yes, 2=no, 9=NA	P1VENTIL	Numeric	1
		IV fluid: 1=yes, 2=no, 9=NA	P1IVFLUID	Numeric	1
		Drugs/pills/medications: 1=yes, 2=no, 9=NA	P1DRUGS	Numeric	1
		Other treatment (specify)	P1OTHTRT	Text	30
**MCH Document / ANC Card**					
109	Weight of baby (gram)<br>(not applicable for miscarriage/abortion)	xxxx grams, 8888=DK, 9999=NA	P1BWEIGHT	Numeric	4
109.1	Weight of baby 2 (gram)<br><br>Continue with 109.2 and P1BWEIGHT3 etc. if needed	xxxx grams, 8888=DK, 9999=NA	P1BWEIGHT2	Numeric	4
110	Weight of woman (kg)	xx.x-yy.y, 99.9=NA	P1FCWEIGHT	Numeric	3
111	Height of woman (cm)	xxx-yyy, 999=NA	P1FCHEIGHT	Numeric	3
112	LMP	01/08/2009 to 30/04/2013 (sites to specify date range), 08/08/1908=don't know, 09/09/1909=NA	P1FCLMP	Date	8
113	Number of antenatal visits	xx-yy, 99=NA	P1FCNUMANC	Numeric	2

POSTNATAL 1 FORM (1-6 days after delivery)					
**No.**	**Question**	**Outcomes**	**Variable Name**	**Field Type**	**Size**
114	Estimated date of delivery	01/08/2009 to 30/04/2013 (sites to specify date range), 08/08/1908=don't know, 09/09/1909=NA	P1FCDDELIV	Date	8
115	Number of Admissions	xx-yy, 99=NA	P1FCNUMADM	Numeric	2
116	Treatments	Hysterectomy: 1=yes, 2=no, 9=NA	P1FCHYSTER	Numeric	1
		Laparotomy or other surgery: 1=yes, 2=no, 9=NA	P1FCSURG	Numeric	1
		Blood transfusion: 1=yes, 2=no, 9=NA	P1FCBLDTRN	Numeric	1
		Assisted breathing: 1=yes, 2=no, 9=NA	P1FCVENTIL	Numeric	1
		IV fluid: 1=yes, 2=no, 9=NA	P1FCIVFLUID	Numeric	1
		Drugs/pills/medications: 1=yes, 2=no, 9=NA	P1FCDRUGS	Numeric	1
		Other treatment (specify)	P1FCOTHER	Text	30

POSTNATAL 2 FORM (42-60 days after delivery)					
**No.**	**Question**	**Outcomes**	**Variable Name**	**Field Type**	**Size**
1.	Date of filling the form<br>(all dates dd/mm/yyyy)	01/06/2010 to 30/04/2013<br>(sites to specify date range)	P2DATEVISIT	Date	8
2.	Study site	11=Bangladesh, 12=DRC, 13=Ghana, 15=India Shivgarh, 16=Kenya, 17=Pakistan Karachi, 18=Pakistan Matiari, 20=Tanzania Pemba, 21=Zambia	SITE	Numeric	2
3.	Worker code	Each site to define valid range	FW	Text	3
4.	Woman ID	Each site to define valid range	WHOWID	Text	10
5	Did the mother deliver more than one child	1=yes, 2=no		Numeric	1
6	If yes, how many?	x		Numeric	1
7	How many of these were live born?	x		Numeric	1
8	Baby ID (only for live births)	Each site to define valid range, 99=NA	WHOBID	Text	10
8.1	Baby ID 2 (only for live births)<br><br>Continue with 8.2 and WHOBID3 etc. if needed	Each site to define valid range, 99=NA	WHOBID2	Text	10
9	Live births/stillbirth:<br>Date of birth<br><br>Miscarriage/abortion:<br>Date of miscarriage/abortion	Each site to define valid range	P2DATEB	Date	8
10	Was the mother available for interview?	11=present, 12=currently in hospital, 13=temporarily absent, 14=died, 15=permanently moved out, 16=refused temporarily, 17=withdrawn consent	P2WSTATUS	Numeric	2
11	If visit could not be made, specify the reason	Text	P2M_REASON	Text	50
12	If mother died, date of death	01/06/2010 to 30/04/2013 (sites to specify date range), 08/08/1908=don't know, 09/09/1909=NA	MDATEDIED	Date	8

POSTNATAL 2 FORM (42-60 days after delivery)					
**No.**	**Question**	**Outcomes**	**Variable Name**	**Field Type**	**Size**
13	Baby's status at time of interview	Live births:<br>11=present, 12=currently in hospital, 13=temporarily absent, 14=died, 15=permanently moved out, 16=refused temporarily, 17=withdrawn consent, 18=visit could not be made<br><br>Other:<br>88=miscarriage/abortion, 99=baby stillborn	P2BSTATUS	Numeric	2
13.1	Baby 2's status at time of interview<br><br>Continue with 13.2 and P2BSTATUS3 etc. if needed	Live births:<br>11=present, 12=currently in hospital, 13=temporarily absent, 14=died, 15=permanently moved out, 16=refused temporarily, 17=withdrawn consent, 18=visit could not be made<br><br>Other:<br>88=miscarriage/abortion, 99=baby stillborn	P2BSTATUS2		
14	If visit could not be made, specify the reason	Text	P2B_REASON	Text	50
14.1	If visit could not be made for baby 2, specify the reason<br><br>Continue with 14.2 and P2B_REASON3 etc. if needed	Text	P2B_REASON2	Text	50
15	If baby died, date of death<br>(not applicable for miscarriage/abortion)	01/06/2010 to 30/04/2013 (sites to specify date range), 08/08/1908=don't know, 09/09/1909=NA	BDATEDIED	Date	8
15.1	If baby 2 died, date of death<br><br>Continue with 15.2 and BDATEDIED3 etc. if needed	01/06/2010 to 30/04/2013 (sites to specify date range), 08/08/1908=don't know, 09/09/1909=NA	BDATEDIED2	Date	8
16	How would you rate your health today?	11=excellent, 12=moderate, 13=poor	P2GHEALTH	Numeric	2

POSTNATAL 2 FORM (42-60 days after delivery)					
**No.**	**Question**	**Outcomes**	**Variable Name**	**Field Type**	**Size**
17	Since the last visit, were you told that you had a hemorrhage?	1=yes, 2=no, 8=don't know	P2TLDHEMO	Numeric	1
18	Since the last visit, have you had any bleeding from the vagina?	1=yes, 2=no, 8=don't know	P2BLEED	Numeric	1
19	Did the bleeding last for longer than one day?	1=yes, 2=no, 8=don't know, 9=NA	P2LBLEED	Numeric	1
20	Did the bleeding wet your clothes, the bed or the floor?	1=yes, 2=no, 8=don't know, 9=NA	P2SEVBLEED	Numeric	1
21	Was the blood fresh (bright red) compared to dark red and viscous?	1=fresh, 2=not fresh, 8=don't know, 9=NA	P2FRESHBLD	Numeric	1
22	Did you lose consciousness around the time of or because of the bleeding?	1=yes, 2=no, 8=don't know, 9=NA	P2BLDCON	Numeric	1
23	Since the last visit, were you told that you have high blood pressure?	1=yes, 2=no, 8=don't know	P2BPTOLD	Numeric	1
24	Measure systolic blood pressure and record results	xxx-yyy, 888=don't know, 999=NA	P2BPSYS	Numeric	3
25	Measure diastolic blood pressure and record results	xxx-yyy, 888=don't know, 999=NA	P2BPDIAS	Numeric	3
26	Measure proteinuria using dipstick and record result	1=2+, 2=1+, 3=normal, 8=don't know, 9=NA	P2DIPSTICK	Numeric	1
27	Since the last visit, did you have a headache?	1=yes, 2=no, 8=don't know	P2HEAD	Numeric	1
28	For how long did you have a headache?	xx-yy days, 88=don't know, 99=NA	P2LHEAD	Numeric	2
29	Was the headache severe?	1=yes, 2=no, 8=don't know, 9=NA	P2SEVHEAD	Numeric	1
30	Since the last visit, did you have any swelling?	1=yes, 2=no, 8=don't know	P2SWELL	Numeric	1
31	For how long did you have the swelling?	xx-yy days, 88=don't know, 99=NA	P2LSWELL	Numeric	2

POSTNATAL 2 FORM (42-60 days after delivery)					
**No.**	**Question**	**Outcomes**	**Variable Name**	**Field Type**	**Size**
32	Was the swelling on your whole body?	1=yes, 2=no, 8=don't know, 9=NA	P2BDYSWELL	Numeric	1
33	Was the swelling on your joints?	1=yes, 2=no, 8=don't know, 9=NA	P2JNTSWELL	Numeric	1
34	Was the swelling on your ankles?	1=yes, 2=no, 8=don't know, 9=NA	P2ANKSWELL	Numeric	1
35	Was the swelling on your face? (Did you have a puffy face?)	1=yes, 2=no, 8=don't know, 9=NA	P2FACSWELL	Numeric	1
36	If the swelling was on any other part of the body, please specify where	Text	P2OTHSWELL	Text	15
37	Since the last visit, did you have blurred vision?	1=yes, 2=no, 8=don't know	P2VISION	Numeric	1
38	For how long did you have blurred vision?	xx-yy days, 88=don't know, 99=NA	P2LVISION	Numeric	2
39	Since the last visit have you had convulsions?	1=yes, 2=no, 8=don't know	P2CONVUL	Numeric	1
40	For how long did you have convulsions?	xx-yy days, 88=don't know, 99=NA	P2LCONVUL	Numeric	1
41	Did you lose consciousness because of the convulsions?	1=yes, 2=no, 8=don't know, 9=NA	P2CONVCON	Numeric	1
42	Since the last visit, did you experience continuously dripping urine?	1=yes, 2=no, 8=don't know	P2URINDRIP	Numeric	1
43	Since the last visit, did you ever lose urine during sudden physical exertion, lifting, coughing or sneezing?	1=yes, 2=no, 8=don't know	P2URINLOSE	Numeric	1
44	Since the last visit, did you ever experience such a strong and sudden urge to urinate that you leak before reaching the toilet?	1=yes, 2=no, 8=don't know	P2URINLEAK	Numeric	1
45	Since the last visit, have you experienced feces passing through the birth canal?	1=yes, 2=no, 8=don't know	P2FECLEAK	Numeric	1
46	Since the last visit, has any physician or healthcare provider told you that you have a medical problem involving your	1=yes, 2=no, 8=don't know	P2VAGPROB	Numeric	1

POSTNATAL 2 FORM (42-60 days after delivery)					
**No.**	**Question**	**Outcomes**	**Variable Name**	**Field Type**	**Size**
	vagina/pelvis or bladder while not pregnant?				
47	Since the last visit, have you undergone surgery in the vagina/pelvis or bladder not including cesarean section, abortion, or surgery for contraception?	1=yes, 2=no, 8=don't know	P2VAGSURG	Numeric	1
48	Were you in a facility when any of these symptoms occurred?	1=yes, 2=no, 8=don't know	P2FACSYMP	Numeric	1
49	If you were at a facility when you experienced any of these symptoms, what type of facility was it?	11=government hospital, 12=government clinic/health centre, 13=private hospital, 14=private clinic/health centre, 66=other, 88=don't know, 99=NA	P2FCTYPSYM	Numeric	2
50	If you were at a facility when you experienced any of these symptoms, were you referred to another facility?	1=yes, 2=no, 8=don't know	P2FACREF	Numeric	1
51	If you were at a facility when you experienced any of these symptoms and were referred, what type of facility was it?	11=government hospital, 12=government clinic/health center, 13=private hospital, 14=private clinic/health center, 66=other, 88=don't know, 99=NA	P2FCREFTYP	Numeric	2
52	If you were at a facility when you experienced any of these symptoms and were referred, did you go to the referral facility?	1=yes, 2=no, 8=don't know, 9=NA	P2FACREFGO	Numeric	1
53	If you were not at a facility when you experience any of these symptoms, did you seek care?	1=yes, 2=no, 9=NA	P2SYMPCSK	Numeric	1
54	If you sought care for any of the symptoms, for which symptoms did you seek care?	Bleeding: 1=yes, 2=no, 9=NA	P2BLDSK	Numeric	1
		High blood pressure: 1=yes, 2=no, 9=NA	P2HBPSK	Numeric	1
		Severe headache: 1=yes, 2=no, 9=NA	P2HEADSK	Numeric	1
		Swelling: 1=yes, 2=no, 9=NA	P2SWELLSK	Numeric	1
		Blurred vision: 1=yes, 2=no, 9=NA	P2VISIONSK	Numeric	1

POSTNATAL 2 FORM (42-60 days after delivery)					
**No.**	**Question**	**Outcomes**	**Variable Name**	**Field Type**	**Size**
		Convulsions: 1=yes, 2=no, 9=NA	P2CONVSK	Numeric	1
		Loss of consciousness: 1=yes, 2=no, 9=NA	P2LCSK	Numeric	1
		Leakage of urine or feces: 1=yes, 2=no, 9=NA	P2LEAKSK	Numeric	1
55	If you sought care what was the first place where you sought care?	11=government hospital, 12=government clinic/health center, 13=private hospital, 14=private clinic/health center, 15=community health worker, 16=traditional healer, 17=pharmacy/druggist, 18=traditional birth attendant, 66=other, 99=NA	P2PLCCSK	Numeric	2
56	Where you admitted to sleep at a facility because of the symptoms you experienced?	1=yes, 2=no, 9=NA	P2ADMIN	Numeric	1
57	For how many nights were you admitted?	11=one day only, 12=1-3 days total, 13=4-6 days total, 14=more than 6 days total, 88=don't know, 99=NA	P2NUMADM	Numeric	2
58	If you sought care, did you receive any of the following treatments?	Hysterectomy (operation to remove your womb): 1=yes, 2=no, 9=NA	P2HYSTEREC	Numeric	1
		Laparotomy or other surgery (any operation where they put you to sleep and operated on your abdomen): 1=yes, 2=no, 9=NA	P2SURGERY	Numeric	1
		Blood transfusion: 1=yes, 2=no, 9=NA	P2BLDTRANS	Numeric	1
		Assisted breathing (by hand or by machine): 1=yes, 2=no, 9=NA	P2VENTIL	Numeric	1
		IV fluid: 1=yes, 2=no, 9=NA	P2IVFLUID	Numeric	1
		Drugs/pills/medications: 1=yes, 2=no, 9=NA	P2DRUGS	Numeric	1
		Other treatment (specify)	P2OTHTRTMT	Text	30

POSTNATAL 2 FORM (42-60 days after delivery)					
**No.**	**Question**	**Outcomes**	**Variable Name**	**Field Type**	**Size**
**MCH Document / ANC Card**					
59	Weight of baby (gram)<br>(not applicable for miscarriage/abortion)	xxxx grams, 8888=DK, 9999=NA	P2BWEIGHT	Numeric	4
59.1	Weight of baby 2 (gram)<br><br>Continue with 59.2 and P2BWEIGHT3 etc. if needed	xxxx grams, 8888=DK, 9999=NA	P2BWEIGHT2	Numeric	4
60	Weight of woman (kg)	xx.x-yy.y, 99.9=NA	P2FCWEIGHT	Numeric	3
61	Height of woman (cm)	xxx-yyy, 999=NA	P2FCHEIGHT	Numeric	3
62	LMP	01/08/2009 to 30/04/2013 (sites to specify date range), 08/08/1908=don't know, 09/09/1909=NA	P2FCLMP	Date	8
63	Number of antenatal visits	xx-yy, 99=NA	P2FCNUMANC	Numeric	2
64	Estimated date of delivery	01/08/2009 to 30/04/2013 (sites to specify date range), 08/08/1908=don't know, 09/09/1909=NA	P2FCDDELIV	Date	8
65	Number of Admissions	xx-yy, 99=NA	P2FCNUMADM	Numeric	2
66	Treatments	Hysterectomy: 1=yes, 2=no, 9=NA	P2FCHYSTER	Numeric	1
		Laparotomy or other surgery: 1=yes, 2=no, 9=NA	P2FCSURG	Numeric	1
		Blood transfusion: 1=yes, 2=no, 9=NA	P2FCBLDTRAN	Numeric	1
		Assisted breathing: 1=yes, 2=no, 9=NA	P2FCVENTIL	Numeric	1
		IV fluid: 1=yes, 2=no, 9=NA	P2FCIVFLUID	Numeric	1
		Drugs/pills/medications: 1=yes, 2=no, 9=NA	P2FCDRUGS	Numeric	1
		Other treatment (specify)	P2FCOTHER	Text	30

BIRTH ATTENDANT FORM (0-6 days after delivery)					
**No.**	**Question**	**Outcomes**	**Variable Name**	**Field Type**	**Size**
1	Date of filling the form<br>(all dates dd/mm/yyyy)	01/06/2010 to 30/04/2013<br>(sites to specify date range)	BDATEVISIT	Date	8
2	Study site	11=Bangladesh, 12=DRC, 13=Ghana, 15=India Shivgarh, 16=Kenya, 17=Pakistan Karachi, 18=Pakistan Matiari, 20=Tanzania Pemba, 21=Zambia	SITE	Numeric	2
3	Worker code	Each site to define valid range	FW	Text	3
4	Woman ID	Each site to define valid range	WHOWID	Text	10
5	Live births/stillbirth:<br>Date of birth<br><br>Miscarriage/abortion:<br>Date of miscarriage/abortion	Each site to define the valid range	BADATEB	Date	8
6	Respondent	1=TBA, 2=family member, 3=midwife/nurse, 4=doctor, 6=other	BIRTHATTEN	Text	10
**Birth Information**					
7	Did the baby cry, move or breathe after birth?	1=yes, 2=no, 8=don't know	BSTILLBORN	Numeric	1
7.1	For baby 2:<br>Did the baby cry, move or breathe after birth?<br><br>Continue with 7.2 and P1STILLB3 etc. if needed	1=yes, 2=no, 8=don't know, 9=NA	BSTILLBORN2	Numeric	1
8	What was the outcome of the delivery?	1=stillbirth, 2=heartbeat present but did not breathe and died, 3=live birth cried but died within 1 hour, 4=live and well	BBSTATUS	Numeric	1
8.1	What was the outcome of the delivery for baby 2?	1=stillbirth, 2=heartbeat present but did not breathe and died, 3=live birth cried but died	BBSTATUS2	Numeric	1

BIRTH ATTENDANT FORM (0-6 days after delivery)					
**No.**	**Question**	**Outcomes**	**Variable Name**	**Field Type**	**Size**
	Continue with 8.2 and BBSTATUS3 etc. if needed	within 1 hour, 4=live and well			
9	If stillbirth, was it a macerated or fresh stillbirth?	1=macerated, 2=fresh, 8=don't know, 9=NA	BSTILLBIRTH	Numeric	1
9.1	For baby 2: If stillbirth, was it a macerated or fresh stillbirth?<br><br>Continue with 9.2 and BSTILLBIRTH3 etc. if needed	1=macerated, 2=fresh, 8=don't know, 9=NA	BSTILLBIRTH2	Numeric	1
10	If baby died, date of death	01/06/2010 to 30/04/2013 (sites to specify date range), 08/08/1908=don't know, 09/09/1909=NA	BBDATEDIED	Date	8
10.1	If baby 2 died, date of death<br><br>Continue with 10.2 and BBDATEDIED3 etc. if needed	01/06/2010 to 30/04/2013 (sites to specify date range), 08/08/1908=don't know, 09/09/1909=NA	BBDATEDIED2	Date	8
**Labor (only for live births or stillbirths)**					
11	When did the woman's water break?	1=before labor started, 2=after labor started or during delivery, 8=don't know	BWATERBRK	Numeric	1
12	How much time before delivery of the baby did the waters break?	1=<24 hours, 2=24 hours or more, 8=don't know, 9=NA (broke during delivery/did not break before C-section)	BWTRBRKTIM	Numeric	1
13	Were the waters clear?	1=clear, 2=not clear/dark, 8=don't know, 9=NA (waters didn't break)	BWTRCOLOR	Numeric	1
14	How much time before delivery of the baby did the woman's labor pains start?	1=<24 hours, 2=24 hours or more, 8=don't know, 9=NA	BLBRSTART	Numeric	1
**Delivery (only for live births or stillbirths)**					
15	Where did the delivery take	1=hospital, 2=other health facility, 3=home,	BPLACEDELIV	Numeric	1

BIRTH ATTENDANT FORM (0-6 days after delivery)					
**No.**	**Question**	**Outcomes**	**Variable Name**	**Field Type**	**Size**
	place?	6=other (specify), 8=don't know			
16	What type of delivery was it?	1=normal vaginal, 2=assisted vaginal (forceps/vacuum), 3=Cesarean section, 8=don't know	BTYPEDELIV	Numeric	1
17	Which part of the baby came out first?	1=head, 2=bottom, 3=feet, 4=arm/hand, 6=other (specify), 8=don't know, 9=NA (C/S)	BPARTDELIV	Numeric	1
17.1	For baby 2:<br>Which part of the baby came out first<br><br>Continue with 17.2 and BPARTDELIV3 etc. if needed	1=head, 2=bottom, 3=feet, 4=arm/hand, 6=other (specify), 8=don't know, 9=NA (C/S)	BPARTDELIV2	Numeric	1
18	If vaginal delivery, did the umbilical cord come out before the baby?	1=yes, 2=no, 8=don't know, 9=NA	BCORDFIRST	Numeric	1
18.1	For baby 2:<br>If vaginal delivery, did the umbilical cord come out before the baby?<br><br>Continue with 18.2 and BCORDFIRST3 etc. if needed	1=yes, 2=no, 8=don't know, 9=NA	BCORDFIRST2	Numeric	1
19	Where was the cord when the baby came out?	1=not around the baby, 2=around the neck, 3=around the body, 4=around other (specify), 8=don't know, 9=NA	BCORDPLACE	Numeric	1

BIRTH ATTENDANT FORM (0-6 days after delivery)					
**No.**	**Question**	**Outcomes**	**Variable Name**	**Field Type**	**Size**
19.1	For baby 2:<br>Where was the cord when the baby came out?<br><br>Continue with 19.2 and BCORDPLACE3 etc. if needed	1=not around the baby, 2=around the neck, 3=around the body, 4=around other (specify), 8=don't know, 9=NA	BCORDPLACE 2	Numeric	1
20	If you the woman had a cesarean section, did you know before she went into labor that she would have a cesarean section?	1=yes, 2=no, 8=don't know, 9=NA	BKNOWCS	Numeric	1
21	If you woman had a cesarean section, was it because the baby was too big?	1=yes, 2=no, 8=don't know, 9=NA	BCSBBIG	Numeric	1
22	If woman had a cesarean section, was it because the baby was lying in a bad position?	1=yes, 2=no, 8=don't know, 9=NA	BCSBPOS	Numeric	1
23	If woman had a cesarean section, was it because she had a small pelvis?	1=yes, 2=no, 8=don't know, 9=NA	BCSSMALLPV	Numeric	1
24	If woman had a cesarean section, was it because she had a ruptured uterus or your uterus might rupture?	1=yes, 2=no, 8=don't know, 9=NA	BCSURUPT	Numeric	1
25	If woman had a cesarean section, was it because her uterus was too weak?	1=yes, 2=no, 8=don't know, 9=NA	BCSUWEAK	Numeric	1
26	If woman had a cesarean section, was it because of	1=yes, 2=no, 8=don't know, 9=NA	BCSBLEED	Numeric	1

BIRTH ATTENDANT FORM (0-6 days after delivery)					
**No.**	**Question**	**Outcomes**	**Variable Name**	**Field Type**	**Size**
	bleeding?				
27	If woman had a cesarean section, was it because of convulsions?	1=yes, 2=no, 8=don't know, 9=NA	BCSCONVULS	Numeric	1
28	Did the placenta come out within one hour of delivery?	1=yes, 2=no, 8=don't know	BPLACENTA	Numeric	1
29	Did the woman have an episiotomy or did anyone cut her vagina during delivery?	1=yes, 2=no, 8=don't know	BEPISIOTOMY	Numeric	1
30	What was done to the baby immediately after delivery?	1=nothing, 2=rubbed, 3=bag and mask resuscitation, 4=mouth-to-mouth resuscitation, 6=other (specify)	BABYCARE	Numeric	1
30.1	What was done to baby 2 immediately after delivery?<br><br>Continue with 30.2 and BABYCARE3 etc. if needed	1=nothing, 2=rubbed, 3=bag and mask resuscitation, 4=mouth-to-mouth resuscitation, 6=other (specify)	BABYCARE2	Numeric	1
31	How many hours after birth did you first put the baby to the breast?	000 to 096, 888 = don't know, 999= not applicable	BBFSTART	Numeric	3
31.1	How many hours after birth did you first put baby 2 to the breast?<br><br>Continue with 31.2 and BBFSTART3 etc. if needed	000 to 096, 888 = don't know, 999= not applicable	BBFSTART2	Numeric	3
**Hemorrhage (applicable for all)**					
32	During delivery/miscarriage/abortion or immediately after, did the woman	1=yes, 2=no, 8=don't know	BHEMO	Numeric	1

BIRTH ATTENDANT FORM (0-6 days after delivery)					
**No.**	**Question**	**Outcomes**	**Variable Name**	**Field Type**	**Size**
	have a hemorrhage?				
33	During delivery/miscarriage/abortion or immediately after, did the woman have any abnormal bleeding from the vagina?	1=yes, 2=no, 8=don't know	BBLEED	Numeric	1
34	Did the bleeding wet her clothes, the bed or the floor?	1=yes, 2=no, 8=don't know, 9=NA	BSEVBLEED	Numeric	1
35	Was the blood fresh (bright red) compared to dark red and viscous?	1=fresh, 2=not fresh, 8=don't know, 9=NA	BFRESHBLD	Numeric	1
36	Did she lose consciousness around the time of or because of the bleeding?	1=yes, 2=no, 8=don't know, 9=NA	BBLDCON	Numeric	1
**Hypertensive Disorders of Pregnancy (applicable for all)**					
37	During delivery/miscarriage/abortion or immediately after, did the woman have a hypertensive disorder?	1=yes, 2=no, 8=don't know	BHYPERT	Numeric	1
38	During delivery/miscarriage/abortion or immediately after, did the woman have high blood pressure?	1=yes, 2=no, 8=don't know	BBPHIGH	Numeric	1
39	Record systolic blood pressure if known	xxx-yyy, 888=don't know, 999=NA	BBPSYS	Numeric	3
40	Record diastolic blood pressure if known	xxx-yyy, 888=don't know, 999=NA	BBPDIAS	Numeric	3
41	Record proteinuria if known	1=2+, 2=1+, 3=normal, 8=don't know, 9=NA	BDIPSTICK	Numeric	1
42	During delivery/miscarriage/abortion or immediately after, did the woman	1=yes, 2=no, 8=don't know	BSEVHEAD	Numeric	1

BIRTH ATTENDANT FORM (0-6 days after delivery)					
**No.**	**Question**	**Outcomes**	**Variable Name**	**Field Type**	**Size**
	have a severe headache?				
43	During delivery/miscarriage/abortion or immediately after, did the woman have any swelling?	1=yes, 2=no, 8=don't know	BSWELL	Numeric	1
44	Was the swelling on her whole body?	1=yes, 2=no, 8=don't know, 9=NA	BBODYSWELL	Numeric	1
45	Was the swelling on her joints?	1=yes, 2=no, 8=don't know, 9=NA	BJOINTSWELL	Numeric	1
46	Was the swelling on her ankles?	1=yes, 2=no, 8=don't know, 9=NA	BANKLSWELL	Numeric	1
47	Was the swelling on her face? (Did you have a puffy face?)	1=yes, 2=no, 8=don't know, 9=NA	BFACESWELL	Numeric	1
48	If the swelling was on any other part of the body, please specify where	Text	BOTHSWELL	Text	15
49	During delivery/miscarriage/abortion or immediately after, did the woman have blurred vision?	1=yes, 2=no, 8=don't know	BVISION	Numeric	1
50	During delivery/miscarriage/abortion or immediately after, did the woman have convulsions?	1=yes, 2=no, 8=don't know	BCONVULSE	Numeric	1
51	Did the woman lose consciousness because of the convulsions?	1=yes, 2=no, 8=don't know, 9=NA	BCONVCON	Numeric	1
**Sepsis (applicable for all)**					
52	Just before or after delivery/miscarriage/abortion, did the woman experience sepsis?	1=yes, 2=no, 8=don't know	BSEPSIS	Numeric	1

BIRTH ATTENDANT FORM (0-6 days after delivery)					
**No.**	**Question**	**Outcomes**	**Variable Name**	**Field Type**	**Size**
53	During delivery/miscarriage/abortion or immediately after, did the woman feel cold to the touch?	1=yes, 2=no, 8=don't know	BCOLD	Numeric	1
54	During delivery/miscarriage/abortion or immediately after, did the woman have a fever?	1=yes, 2=no, 8=don't know	FEVER	Numeric	1
55	Just before or after delivery/miscarriage/abortion, did the woman have smelly discharge or pus pass from her vagina?	1=yes, 2=no, 8=don't know	BVDISCHG	Numeric	1
**Fistula (applicable for all)**					
56	During delivery/miscarriage/abortion or immediately after, did the woman have leakage or urine not related to delivery?	1=yes, 2=no, 8=don't know	BURINELEAK	Numeric	1
57	During delivery/miscarriage/abortion or immediately after, did the woman have feces passing through the birth canal (not related to delivery)?	1=yes, 2=no, 8=don't know	BFECESLEAK	Numeric	1
58	Does the woman have a fistula?	1=yes, 2=no, 8=don't know	BFISTULA	Numeric	1
**Treatment**					
59	Did you recommend that the woman receive any of the following treatments / did you perform or give any of the following?	Hysterectomy (operation to remove your womb): 1=yes, 2=no, 9=NA	BHYSTEREC	Numeric	1
		Laparotomy or other surgery (any operation where they put you to sleep and operated on your abdomen): 1=yes, 2=no, 9=NA	BSURGERY	Numeric	1

BIRTH ATTENDANT FORM (0-6 days after delivery)					
**No.**	**Question**	**Outcomes**	**Variable Name**	**Field Type**	**Size**
		Tear in vagina repaired: 1=yes, 2=no, 9=NA	BREPTEARVG	Numeric	1
		Blood transfusion: 1=yes, 2=no, 9=NA	BBLDTRANS	Numeric	1
		Assisted breathing (by hand or by machine): 1=yes, 2=no, 9=NA	BVENTILATE	Numeric	1
		IV fluid: 1=yes, 2=no, 9=NA	BIVFLUID	Numeric	1
		Drugs/pills/medications: 1=yes, 2=no, 9=NA	BDRUGS	Numeric	1
		Other treatment (specify)	BOTHTRTMT	Text	30

FACILITY SURVEILLANCE FORM					
**No.**	**Question**	**Outcomes**	**Variable Name**	**Field Type**	**Size**
	Date of filling the form<br>(all dates dd/mm/yyyy)	01/06/2010 to 30/04/2013<br>(sites to specify date range)	FDATEVISIT	Date	8
	Study site	11=Bangladesh, 12=DRC, 13=Ghana, 15=India Shivgarh, 16=Kenya, 17=Pakistan Karachi, 18=Pakistan Matiari, 20=Tanzania Pemba, 21=Zambia	SITE	Numeric	2
	Worker code	Each site to define valid range	FW	Text	3
	Woman ID	Each site to define valid range	WHOWID	Text	10
	Baby ID	Each site to define valid range	WHOBID	Text	10
	Date of birth	Each site to define valid range	FDATEB	Date	8
	Number of deliveries	xx-yy, 99=NA	FNUMDELIV	Numeric	2

FACILITY SURVEILLANCE FORM					
**No.**	**Question**	**Outcomes**	**Variable Name**	**Field Type**	**Size**
	What type of delivery was it?	1=normal vaginal, 2=assisted vaginal (forceps/vacuum), 3=Cesarean section, 9=NA	FTYPEDELIV	Numeric	1
**Admissions with Obstetrical Complications**					
	Did the woman have any abortion complications?	1=yes, 2=no, 9=NA	FCABORTION	Numeric	1
	Did the woman have an antepartum hemorrhage?	1=yes, 2=no, 9=NA	FANTEHEMO	Numeric	1
	Did the woman have gestational hypertensive disorder?	1=yes, 2=no, 9=NA	FGESTHTN	Numeric	1
	Did the woman have pre-eclampsia?	1=yes, 2=no, 9=NA	FPREECLAMP	Numeric	1
	Did the woman have eclampsia?	1=yes, 2=no, 9=NA	FECLAMP	Numeric	1
	Did the woman have sepsis?	1=yes, 2=no, 9=NA	FOBSEPSIS	Numeric	1
	Did the woman have any other obstetrical complications?	1=yes (specify), 2=no, 9=NA	FOTHOBCOMP	Numeric	1
1	If the woman had any of the above obstetrical complications, were they resolved?	Abortion complications: 1=resolved, 2=improved, 3=died, 8=DK, 9=NA	FRESABORT	Numeric	1
		Antepartum hemorrhage: 1=resolved, 2=improved, 3=died, 8=DK, 9=NA	FRESAHEMO	Numeric	1
		Gestational hypertensive disorder: 1=resolved, 2=improved, 3=died, 8=DK, 9=NA	FRESGESHTN	Numeric	1
		Pre-eclampsia: 1=resolved, 2=improved, 3=died, 8=DK, 9=NA	FRESPREECL	Numeric	1
		Eclampsia: 1=resolved, 2=improved, 3=died, 8=DK, 9=NA	FRESECLAMP	Numeric	1
		Sepsis: 1=resolved, 2=improved, 3=died, 8=DK, 9=NA	FRESSEPSIS	Numeric	1
		Other: 1=resolved, 2=improved, 3=died, 8=DK, 9=NA	FRESOBOTH	Numeric	1

FACILITY SURVEILLANCE FORM					
**No.**	**Question**	**Outcomes**	**Variable Name**	**Field Type**	**Size**
2	If the woman had any of the above obstetrical complications, were any of the following treatments given?	Hysterectomy: 1=yes, 2=no, 8=DK, 9=NA	FOBHYSTER	Numeric	1
		Laparotomy or other surgery: 1=yes, 2=no, 9=NA, 8=DK,	FOBSURGERY	Numeric	1
		Blood transfusion: 1=yes, 2=no, 8=DK, 9=NA	FOBBLDTRAN	Numeric	1
		Assisted breathing (by hand or by machine): 1=yes, 2=no, 8=DK, 9=NA	FOBVENTIL	Numeric	1
		IV fluid: 1=yes, 2=no, 9=NA, 8=DK,	FOBIVFLUID	Numeric	1
		Drugs/pills/medications: 1=yes, 2=no, 9=NA, 8=DK,	FOBDRUGS	Numeric	1
3	If there was a blood transfusion, record the following	Whole blood:<br>11=0 units<br>12=1 units<br>13=2 units<br>14=3 units and more<br>88= D/K	FWHOLEBLD	Numeric	1
		PRBC: xxx-yyy mL, 999=NA, 888=DK	FPRBC	Numeric	3
		FFP: xxx-yyy mL, 999=NA, 888=DK	FFFP	Numeric	3
		Platelets: xx-yy units, 99=NA, 88=DK	FPLATELETS	Numeric	2
**Delivery Complications**					
4	Did the woman have an intrapartum hemorrhage?	1=yes, 2=no, 9=NA	FINTRAHEMO	Numeric	1
5	Did the woman have a postpartum hemorrhage?	1=yes, 2=no, 9=NA	FPOSTHEMO	Numeric	1
6	Did the woman have primary sepsis?	1=yes, 2=no, 9=NA	FPRSEPSIS	Numeric	1
7	Did delivery involve a breech or malposition of the baby?	1=yes, 2=no, 9=NA	FDELPOSN	Numeric	1
8	Was the cord wrapped around the	1=yes, 2=no, 9=NA	FCORDWRAP	Numeric	1

FACILITY SURVEILLANCE FORM					
**No.**	**Question**	**Outcomes**	**Variable Name**	**Field Type**	**Size**
	baby's neck?				
9	Was the labor prolonged?	1=yes, 2=no, 9=NA	FPROLABOR	Numeric	1
10	Was there a prolonged rupture of membranes?	1=yes, 2=no, 9=NA	FPRORUPT	Numeric	1
11	Was there a retained placenta?	1=yes, 2=no, 9=NA	FPLACENTA	Numeric	1
12	Did the woman have a perianal tear?	1=yes, 2=no, 9=NA	FPERITEAR	Numeric	1
13	Did the woman have any other delivery complications?	1=yes (specify), 2=no, 9=NA	FOTHDELC	Numeric	1
14	If the woman had any of the above delivery complications, were they resolved?	Intrapartum hemorrhage: 1=resolved, 2=improved, 3=woman died, 4=baby died, 9=NA	FRESIHEMO	Numeric	1
		Postpartum hemorrhage: 1=resolved, 2=improved, 3=woman died, 4=baby died, 9=NA	FRESPHEMO	Numeric	1
		Primary sepsis: 1=resolved, 2=improved, 3=woman died, 4=baby died, 9=NA	FRESPSEPSIS	Numeric	1
		Breech/malposition: 1=resolved, 2=improved, 3=woman died, 4=baby died, 9=NA	FRESDELPOS	Numeric	1
		Cord wrapped around neck: 1=resolved, 2=improved, 3=woman died, 4=baby died, 9=NA	FRESCORDW	Numeric	1
		Prolonged labor: 1=resolved, 2=improved, 3=woman died, 4=baby died, 9=NA	FRESPLABOR	Numeric	1
		Prolonged rupture of membranes: 1=resolved, 2=improved, 3=woman died, 4=baby died, 9=NA	FRESPRUPT	Numeric	1
		Retained placenta: 1=resolved, 2=improved, 3=woman died, 4=baby died, 9=NA	FRESPLACEN	Numeric	1
		Perianal tear: 1=resolved, 2=improved,	FRESPTEAR	Numeric	1

FACILITY SURVEILLANCE FORM					
**No.**	**Question**	**Outcomes**	**Variable Name**	**Field Type**	**Size**
		3=woman died, 4=baby died, 9=NA			
		Other delivery complications: 1=resolved, 2=improved, 3=woman died, 4=baby died, 9=NA	FRESCDOTH	Numeric	1
15	If the woman had any of the above delivery complications, were any of the following treatments given?	Hysterectomy: 1=yes, 2=no, 9=NA	FDBHYSTER	Numeric	1
		Laparotomy or other surgery: 1=yes, 2=no, 9=NA	FDBSURGERY	Numeric	1
		Blood transfusion: 1=yes, 2=no, 9=NA	FDBBLDTRAN	Numeric	1
		Assisted breathing (by hand or by machine): 1=yes, 2=no, 9=NA	FDBVENTIL	Numeric	1
		IV fluid: 1=yes, 2=no, 9=NA	FDBIVFLUID	Numeric	1
		Drugs/pills/medications: 1=yes, 2=no, 9=NA	FDBDRUGS	Numeric	1
16	If there was a blood transfusion, record the following	Whole blood:<br>11=0 units<br>12=1 units<br>13=2 units<br>14=3 units and more<br>88= D/K	FWHOLEBLD_DC	Numeric	1
		PRBC: xxx-yyy mL, 999=NA, 888=DK	FPRBC_DC	Numeric	3
		FFP: xxx-yyy mL, 999=NA, 888=DK	FFFP_DC	Numeric	3
		Platelets: xx-yy units, 99=NA, 88=DK	FPLATELETS_DC	Numeric	2
**Admissions for Postpartum Complications**					
17	Did the woman have sepsis?	1=yes, 2=no, 9=NA	FPPSEPSIS	Numeric	1
18	Did the woman have secondary postpartum hemorrhage?	1=yes, 2=no, 9=NA	FSPOSTHEMO	Numeric	1
19	Did the woman have a fistula?	1=yes, 2=no, 9=NA	FFISTULA	Numeric	1
20	Did the woman have any other postpartum complications for which she was admitted?	1=yes, 2=no, 9=NA	FPPOTH	Numeric	1

FACILITY SURVEILLANCE FORM					
**No.**	**Question**	**Outcomes**	**Variable Name**	**Field Type**	**Size**
**Testing Results**					
21	Systolic blood pressure	xxx-yyy, 999=NA	FBPSYS	Numeric	3
22	Diastolic blood pressure	xxx-yyy, 999=NA	FBPDIAS	Numeric	3
23	Proteinuria	1=2+, 2=1+, 3=normal, 9=NA	FDIPSTICK	Numeric	1
24	Malaria RDT / blood smear	1=positive, 2=negative, 9=NA	FMRDT	Numeric	1
25	Hemoglobin	xx.x-yy.y g/dL, 99.9=NA	FHEMOGLOB	Numeric	3
26	Hematocrit	xx-yy %, 99=NA	FHEMATOCR	Numeric	2

BASELINE FORM (completed at the first antenatal visit)					
**No.**	**Question**	**Outcomes**	**Variable Name**	**Field Type**	**Size**
1.	Date of filling the form (all dates dd/mm/yyyy)	01/06/2012 to 30/04/2013	DATEVISIT	Date	8
2.	Study site	11=Bangladesh, 12=DRC, 13=Ghana, 15=India Shivgarh, 16=Kenya, 17=Pakistan Karachi, 18=Pakistan Matiari, 20=Tanzania Pemba, 21=Zambia	SITE	Numeric	2
3.	Worker code	Each site to define valid range	FW	Text	3
4.	Woman ID	Each site to define valid range	WHOID	Text	10
5.	Has the woman given her consent to be enrolled in the Cohort study?	1=yes, 2=no	CONSENT	Numeric	2
**PREGNANCY HISTORY**					
6.	Including this pregnancy, how many times in your life have you been pregnant?	xx-yy, 88=don’t know	GRAVIDITY	Numeric	2

BASELINE FORM (completed at the first antenatal visit)					
**No.**	**Question**	**Outcomes**	**Variable Name**	**Field Type**	**Size**
7.	How many deliveries of a live child have you had?	xx-yy, 88=don't know; 99=NA, first pregnancy	PARITY	Numeric	2
8.	How many deliveries of a stillborn child have you had (e.g., the baby did not cry or move or breathe after delivery)	xx-yy, 88=don't know; 99=NA, first pregnancy	PREVSB	Numeric	2
9.	How many pregnancies have you had which ended before 6 months?	xx-yy, 88=don't know; 99=NA, first pregnancy	PREVMIS	Numeric	2
10.	How many living children do you have?	xx-yy, 88=don't know; 99=NA, first pregnancy	ALIVECHILD	Numeric	2
11.	How many of your children have died?	xx-yy, 88=don't know; 99=NA, first pregnancy or no live born children	DIEDCHILD	Numeric	2
12.	Did you attend ANC for all, some or none of your previous pregnancies?	11=all; 12=some; 13=none; 99=NA, first pregnancy	PREVANCSK	Numeric	2
13.	In any of your previous pregnancies, did you have severe bleeding or haemorrhaging before delivery?	1=yes, 2=no; 9=NA, first pregnancy	PREVHEMOAP	Numeric	1
14.	In any of your previous pregnancies, did you have severe bleeding or haemorrhaging after delivery?	1=yes, 2=no; 9=NA, first pregnancy	PREVHEMOPP	Numeric	1
15.	In any of your previous pregnancies, did you have fits or convulsions?	1=yes, 2=no; 9=NA, first pregnancy	PREVCONV	Numeric	1
16.	In any of your previous pregnancies, did your water break more than 24 hours (one day) before labour pains started?	1=yes, 2=no; 9=NA, first pregnancy	PREVPROM	Numeric	1

BASELINE FORM (completed at the first antenatal visit)					
**No.**	**Question**	**Outcomes**	**Variable Name**	**Field Type**	**Size**
17.	In any of your previous pregnancies, were you admitted to stay overnight in the hospital three or more nights?	1=yes, 2=no; 9=NA, first pregnancy	PREVPADM	Numeric	1
18.	In any of your previous pregnancies, did you deliver twins or triplets?	1=yes, 2=no; 9=NA, first pregnancy	PREVMULTIP	Numeric	1
19.	In any of your previous pregnancies, did you have a caesarean section?	1=yes, 2=no; 9=NA, first pregnancy	PREVCS	Numeric	1
20.	Did any of your previous pregnancies end more than 1 month before time?	1=yes, 2=no; 9=NA, first pregnancy	PREVPREM	Numeric	1
21.	In any of your previous pregnancies, have any other serious problem that I haven't asked you about?	1=yes, 2=no; 9=NA, first pregnancy	PREVOTHER	Numeric	1
22.	If yes, other serious problems, specify:	Text	SPREVOTHER	Text	50
23.	In the year before THIS pregnancy, did you have any health problems?	1=yes, 2=no	PROBBFPREG	Numeric	1
24.	If yes, health problems in year before pregnancy, specify:	Text	SPROBBF	Text	50
25.	Has a doctor every told you that you have a medical problem involving your private parts/pelvis or bladder while not pregnant?	1=yes, 2=no	PREVFIST	Numeric	1

BASELINE FORM (completed at the first antenatal visit)					
**No.**	**Question**	**Outcomes**	**Variable Name**	**Field Type**	**Size**
26.	Have you ever undergone surgery in the private parts, pelvis or bladder, not related to pregnancy (i.e. surgery other than caesarean section, abortion or surgery for contraception)?	1=yes, 2=no	OREVOPFIS	Numeric	1
27.	Have you ever had a fit / convulsion when you were not pregnant?	1=yes, 2=no	NPREGCONV	Numeric	1
28.	Has a doctor ever told you that you have epilepsy?	1=yes, 2=no	EPILEPSY	Numeric	1
**BACKGROUND DETAILS MOTHER**					
29.	What is your age?	xx-yy, 88=don't know	MOTHERAGE	Numeric	2
30.	Place woman in an age category:	11=15-19 years; 12=20-24 years; 13=25-29 years; 14=30=34 years; 15=35-39 years; 16=40-44 years; 17=45-49 years.	ATTENDANT1	Numeric	2
31.	How many years of schooling did you complete?	00 to 25, 99=not applicable	MOTHEREDU	Numeric	2
32.	What is your current occupation?	11= government job, 12= private job, 13= self employed, 14= farming only, 15= daily wage earner, 16= other work, 17= does not work, , 99=not applicable	MOTHEROCC	Numeric	2
33.	If occupation is "other work", specify	Text	MOCCOTH	Text	50

BASELINE FORM (completed at the first antenatal visit)					
**No.**	**Question**	**Outcomes**	**Variable Name**	**Field Type**	**Size**
**BACKGROUND DETAILS HOUSEHOLD**					
34.	Who is the head of your household?	11= Mother of baby, 12= Father of baby, 13= Grandmother of baby, 14= Grandfather of baby, 15= other	HEAD	Numeric	2
35.	How many years of schooling did the baby's father of the baby or head of the household complete?	00 to 25, 99= not applicable 88= not known	FATHEREDU	Numeric	2
36.	What is the current occupation of the father or the head of the household?	11= government service, 12= private service, 13= daily wage earner, 14= self employed, 15= farming, 16= does not work, 17= other, 99=not applicable	FATHEROCC	Numeric	2
37.	If occupation of baby's father or head of the household is "other", specify	Text	FOCCOTH	Text	20
38.	Is baby's father currently staying with the family?	1=yes, 2= no, 9=not applicable	FATHSTAY	Numeric	1
39.	What is the main source of drinking water for members of your household?	11= piped water into the dwelling, 12= public tap, 13= Tube well or borehole or hand pump, 14= Open well, 15= closed well, 16= Tanker truck, 17= small cart with tank, 18= Surface water (river / dam / lake / pond / stream / canal), 19= bottled water, 20= rain water, 21= other	HOUSEWTR	Numeric	2
40.	If source of water is "other", specify	Text	WTROTHER	Text	20
41.	What kind of toilet facility do you usually use?	11= Flush or pour flush toilet, 12= Pit latrine, 13= dry toilet, 14= bucket latrine, 15= No toilet facility (uses open space or field), 16= other	TOILET	Numeric	2

BASELINE FORM (completed at the first antenatal visit)					
**No.**	**Question**	**Outcomes**	**Variable Name**	**Field Type**	**Size**
42.	If toilet facility is "other", specify	Text	TOILETOTH	Text	20
43.	What is the religion of the head of the household?	11= Christian, 12= Muslim, 13= Hindu, 14= none, 15= Traditional African, 16=other	HHRELIG	Numeric	2
44.	If religion "other", specify	Text	RELIGOTH	Text	20
45.	What is the ethnic group of the head of the household?	**Each site to decide possible outcomes**	**XX_ETHNIC**	Numeric	2
46.	What type of fuel does the household mainly use for cooking	11= electricity, 12= liquid petroleum gas / natural gas, 13= kerosene, 14= coal / lignite, 15= charcoal, 16= wood, 17= straw /shrub / grass, 18= agricultural crop waste, 19= dung cakes, 20= biogas, 21= other	FUELCOOK	Numeric	2
47.	If cooking fuel is "other", specify	Text	FUELOTH	Text	20
48.	How many rooms in your house (including the kitchen) are used for sleeping?	01 to 20	ROOMS	Numeric	2
49.	How many persons slept in the house last night?	01 to 40	PERSONS	Numeric	2
**ASSETS (to be used for PCA in determining wealth quintiles)**<br>**QUESTIONS TO BE DECIDED BY EACH SITE. YOU CAN USE THE COUNTRY DHS SURVEY QUESTIONNAIRE**					

BASELINE FORM (completed at the first antenatal visit)					
**No.**	**Question**	**Outcomes**	**Variable Name**	**Field Type**	**Size**
**SEE EXAMPLE QUESTIONS BELOW FOR GHANA**					
50	Do you own or rent the house you live in, or do you have another type of arrangement, such as “perching”?	11= Sole Ownership, 12= Joint Ownership, 13= Renting, 14= Family/relative’s house, 15= House provided rent free, 16= Perching, 17= Other, 88= NK	GH_OWNHOUSE	Numeric	2
51	Does this household own any land?	1= Yes, 2= no	GH_OWNLAND	Numeric	1
	**Does anyone in the household own:**				
52	Is there electrical supply at home?	1. Yes, 2. No	GH_ELECTR	Numeric	1
53	Chickens	1. Yes, 2. No	GH_CHICKEN	Numeric	1
54	Sheep	1. Yes, 2. No	GH_SHEEP	Numeric	1
55	Other animals	1. Yes, 2. No	GH_OTHANIMAL	Numeric	1
56	Mattress	1. Yes, 2. No	GH_MATTRESS	Numeric	1
57	Stove or cooker	1. Yes, 2. No	GH_PRESCOOK	Numeric	1
58	Chair	1. Yes, 2. No	GH_CHAIR	Numeric	1
59	Cot or bed	1. Yes, 2. No	GH_BED	Numeric	1
60	Divider	1. Yes, 2. No	GH_DIVIDER	Numeric	1

BASELINE FORM (completed at the first antenatal visit)					
**No.**	**Question**	**Outcomes**	**Variable Name**	**Field Type**	**Size**
61	Table	1. Yes, 2. No	GH_TABLE	Numeric	1
62	Electric fan	1. Yes, 2. No	GH_FAN	Numeric	1
63	Radio	1. Yes, 2. No	GH_RADIO	Numeric	1
64	Television	1. Yes, 2. No	GH_BWTV	Numeric	2
65	Sewing machine	1. Yes, 2. No	GH_SEW	Numeric	1
66	Mobile telephone	1. Yes, 2. No	GH_MOBILE	Numeric	1
67	mosquito net	1. Yes, 2. No	GH_MOSNET	Numeric	1
68	Computer	1. Yes, 2. No	GH_COMP	Numeric	1
69	refrigerator	1. Yes, 2. No	GH_FRIDGE	Numeric	1
70	Watch or clock	1. Yes, 2. No	GH_WATCH	Numeric	1
71	Bicycle	1. Yes, 2. No	GH_BICYCLE	Numeric	1
72	Motor cycle or scooter	1. Yes, 2. No	GH_MOTOCY	Numeric	1
73	Animal-drawn cart	1. Yes, 2. No	GH_ANIMCART	Numeric	1
74	Car	1. Yes, 2. No	GH_CAR	Numeric	1
75	Thresher	1. Yes, 2. No	GH_THRSHR	Numeric	1
76	Tractor	1. Yes, 2. No	GH_TRACTOR	Numeric	1

BASELINE FORM (completed at the first antenatal visit)					
**No.**	**Question**	**Outcomes**	**Variable Name**	**Field Type**	**Size**
78	**Materials used in the construction of the house (observe):**				
79	Flooring in sleeping rooms	11=Cement, 12= Mud/clay	GH_FLOOR	Numeric	2
80	Roof of household	11=Metal, 12=Asbestos, 13=Thatch; 14=Mud; 15=Other	GH_ROOF	Numeric	2
81	Wall of household	11=Cement, 12=Mud, 13=Other	GH_WALL	Numeric	2
